# Infrared Treatment Alters the Chemical Profile and In Vitro Bioactivity of Jojoba Oil: Antimicrobial, Biofilm Formation Inhibition and Ovarian Cancer Cell Responses

**DOI:** 10.3390/ijms27219415

**Published:** 2026-10-22

**Authors:** Husam Qanash, Aisha M. H. Al-Rajhi, Abdulrahman S. Bazaid, Fahad Almarshadi, Nourah M. Almimoni, Hussam A. Althagafi, Abdullah M. Almotayri, Reham Yahya, Sohaila Fathi El-Hawary, Abdu Aldarhami

**Affiliations:** 1 Department of Medical Laboratory Science, College of Applied Medical Sciences, University of Ha’il, Hail 55476, Saudi Arabia; 2 Medical and Diagnostic Research Center, University of Ha’il, Hail 55473, Saudi Arabia; 3 Department of Biology, College of Science, Princess Nourah bint Abdulrahman University, P.O. Box 84428, Riyadh 11671, Saudi Arabia; 4 Public Health Department, College of Public Health and Health Informatics, University of Ha’il, Hail 55473, Saudi Arabia; 5 Department of Biology, Faculty of Science, Al-Baha University, Al-Baha 65779, Saudi Arabia; 6 Basic Sciences Department, College of Science and Health Professions, King Saud Bin Abdulaziz University for Health Sciences, Riyadh 11671, Saudi Arabia; 7 King Abduallah International Medical Research Center, P.O. Box 3661, Riyadh 11481, Saudi Arabia; 8 Department of Biology, Collage of Science, Jazan University, P.O. Box 114, Jazan 45142, Saudi Arabia; 9 Department of Microbiology and Parasitology, Faculty of Medicine, Umm Al-Qura University, Al-Qunfudah 28815, Saudi Arabia

**Keywords:** FTIR/GC-MS profiling, thermal control, multidrug-resistant pathogens, *Candida albicans*, apoptosis-related gene expression, fibroblast migration

## Abstract

Jojoba oil (*Simmondsia chinensis*) is widely used in cosmetic and pharmaceutical formulations, yet approaches to enhance its biological activity remain insufficiently explored. This study investigated whether infrared (IR) treatment could modify the chemical profile and in vitro biological properties of jojoba oil. A thermal control matched to the IR temperature–time profile was included to separate bulk heating from IR-specific effects. Crude (untreated) and IR-treated oil (30 min, 50 mW/cm^2^) were characterized by Fourier-transform infrared spectroscopy (FTIR) and gas chromatography-mass spectrometry (GC-MS), followed by evaluation of antimicrobial, antibiofilm, inhibition of protein denaturation, fibroblast scratch-closure, and antiproliferative activity against SK-OV-3 cells. IR treatment altered the FTIR profile and produced substantial changes in the GC-MS-detectable composition, including the detection of a peak tentatively assigned to 9-hexadecenoic acid, 9-octadecenyl ester (27.45% relative peak area), and 9-eicosyne (8.97% relative peak area); these values represent relative chromatographic abundance, not absolute concentrations. Compared with crude oil, IR-treated jojoba oil exhibited two- to fourfold lower minimum inhibitory concentrations (MIC), reaching 7.8 µg/mL against *Candida albicans*, and inhibited *Staphylococcus aureus* biofilm formation by 96.8%. It also enhanced fibroblast scratch closure (84.1% vs. 67.0%) and showed greater inhibition of protein denaturation (IC_50_, 4.43 vs. 6.52 µg/mL). Against SK-OV-3 ovarian cancer cells, IR-treated oil reduced the IC_50_ from 114.5 to 78.7 µg/mL and produced marked G0/G1 accumulation (97.9%). This response was accompanied by increased *BAX* (3.67-fold) and *CASP3* (5.73-fold) expression and decreased *BCL2* expression (0.61-fold). Collectively, these findings demonstrate that IR treatment, beyond a matched thermal control, substantially modifies jojoba oil and enhances multiple in vitro biological activities. The novelty is the linkage between IR-induced chemical alteration and functional biological changes, not the mere combination of assays.

## 1. Introduction

Plant-derived oils have attracted increasing scientific and industrial interest because of their diverse biological activities, natural origin, and potential as alternatives to synthetic compounds in biomedical and food-related applications. These oils contain a broad range of bioactive constituents, including phenolic compounds, terpenes, fatty acids, and antioxidants, which contribute to their antimicrobial, anti-inflammatory, preservative, and health-promoting properties [1,2]. In the food industry, vegetable oils are increasingly explored as natural functional ingredients and preservatives to improve product quality and extend shelf life, whereas in biomedical applications, they have been investigated for their potential roles in disease prevention, wound repair, and the development of biocompatible bioactive formulations [3,4]. Their biological compatibility, accessibility, and environmentally favorable characteristics further support their growing relevance across pharmaceutical, cosmetic, and food-related applications.

Jojoba oil, a liquid wax obtained from the seeds of *Simmondsia chinensis* C.K. Schneid. (Simmondsiaceae), has long been incorporated into dermocosmetic and pharmaceutical formulations because of its distinctive physicochemical stability, skin permeability, and biocompatibility [5,6,7]. Unlike conventional triglyceride-rich vegetable oils, jojoba oil consists predominantly of long-chain wax esters (C36–C46), together with free fatty acids, long-chain alcohols, tocopherols, and other minor constituents. This unusual chemical composition contributes to its oxidative stability and has been associated with anti-inflammatory, antioxidant, and tissue-regenerative properties [8,9]. Nevertheless, the broader biomedical application of crude jojoba oil remains constrained by compositional variability and its relatively moderate antimicrobial and antiproliferative activity against SK-OV-3 cells [10,11]. Accordingly, approaches capable of modifying its chemical profile and potentially enhancing its biological performance, without introducing additional chemical reagents, are of considerable interest.

Physical modification of plant-derived oils using electromagnetic radiation has recently emerged as a potentially attractive green-processing strategy. Infrared (IR) radiation spans wavelengths of approximately 780 nm to 1 mm and can alter molecular interactions and induce physicochemical changes in organic matrices under appropriately controlled conditions [12,13]. Depending on the irradiation conditions and chemical composition of the substrate, such exposure may influence molecular configuration, oxidation-related reactions, and the relative abundance or detectability of individual constituents. These changes could subsequently modify the biological properties of treated oils. However, the effect of IR treatment on jojoba oil itself has not been defined. Previous jojoba studies have characterized wax ester composition, cosmetic performance, antimicrobial/anti-inflammatory potential, and nanoemulsion formulation [2,5,6,7,8,9,14], but none applied IR as a controlled physical modification. Conversely, IR processing has been studied in other plant oils and food matrices [12,13], but the underlying lipid-matrix chemistry and its relevance to jojoba wax esters remain undefined. Thus, the specific gap is twofold: whether a defined IR dose (30 min, 50 mW/cm^2^) produces independently measurable FTIR and GC-MS changes in jojoba oil and whether these chemical changes coincide with reproducible functional shifts in antimicrobial, antibiofilm, protein denaturation, fibroblast scratch-closure, and SK-OV-3 ovarian cancer cell responses. The novelty of this study therefore rests on the IR-induced chemical alteration and its biological correlates, not on the number of bioassays performed.

This question is particularly relevant in the context of antimicrobial resistance and biofilm-associated infections. The increasing prevalence of microorganisms with reduced susceptibility to conventional antimicrobial agents has intensified the search for naturally derived products capable of acting against both planktonic cells and surface-associated microbial communities [15]. Biofilms are particularly challenging because their structured extracellular matrix and altered cellular physiology can substantially reduce susceptibility to antimicrobial treatment. Jojoba oil has demonstrated antimicrobial activity against several microorganisms, including Gram-positive bacteria and *Candida* species, although its activity against robust biofilm-forming organisms such as *Pseudomonas aeruginosa* may be limited [14,16]. Modification of the chemical composition of jojoba oil through IR treatment may therefore alter its antimicrobial and antibiofilm performance, potentially by changing the abundance or availability of constituents capable of interacting with microbial membranes or biofilm-associated processes. Such possibilities, however, require experimental evaluation rather than mechanistic assumption.

The potential value of a multifunctional natural product extends beyond antimicrobial activity. Persistent inflammation, delayed tissue repair, and microbial colonization frequently coexist in difficult-to-heal wounds, creating a need for agents capable of addressing more than one component of the pathological process [17,18]. Jojoba oil is already recognized for its emollient and anti-inflammatory properties and has been investigated for its capacity to support skin integrity and tissue repair [19]. Determining whether IR treatment can modify or enhance these biological effects may therefore provide additional insight into its suitability for future topical or biomaterial-related applications.

The potential antiproliferative activity of modified jojoba oil against ovarian cancer cells also warrants investigation. However, because the present study evaluates only a single cancer cell line and does not include a normal-cell comparator, any findings will be limited to in vitro cytotoxicity/antiproliferative effects and will not establish selective antiproliferative activity against SK-OV-3 cells. Ovarian cancer remains a major cause of gynecological cancer-related mortality, largely because many cases are diagnosed at advanced stages and eventually develop resistance to conventional therapy [20]. Natural products continue to be investigated as sources of bioactive molecules capable of suppressing cancer-cell proliferation and modulating pathways involved in programmed cell death. The intrinsic apoptotic pathway is regulated, in part, by the balance between pro-apoptotic proteins, such as *BAX*, and anti-apoptotic proteins, such as *BCL-2*, with downstream activation of executioner *caspases*, including *caspase-3* [21,22]. In parallel, disruption of normal cell-cycle progression at the G0/G1, S, or G2/M phases can contribute to suppression of tumor-cell proliferation [23,24]. Although crude (untreated) jojoba oil has previously attracted interest in several biological applications, whether IR-treated jojoba oil can influence SK-OV-3 ovarian cancer cell viability, cell-cycle distribution, and apoptosis-associated gene expression has not been systematically examined. The absence of a parallel normal-cell comparator in the present design means that selectivity and therapeutic window cannot be assessed.

Against this background, the present study provides an integrated investigation of the chemical and biological consequences of IR treatment of jojoba oil. The novelty of this work lies in demonstrating that a defined IR treatment produces specific, independently measurable changes in jojoba oil chemistry, attenuation of the 1739 cm^−1^ ester carbonyl band, emergence of a 1633 cm^−1^ band, O–H shift/broadening, reduced CH_2_ rocking, and GC-MS changes, including the appearance of 9-hexadecenoic acid, 9-octadecenyl ester (Z,Z) (27.45%), 9-eicosyne (8.97%), and cis-10-nonadecenoic acid (4.09%), and increased cis-13-eicosenoic acid (6.34% to 18.17%), that are accompanied by enhanced antimicrobial/antibiofilm activity, greater protein denaturation, improved fibroblast scratch closure, and stronger SK-OV-3 antiproliferative and apoptosis-associated responses. This linkage, rather than the parallel use of multiple assays, is the principal novelty. Specifically, this study aimed to characterize IR-associated changes in jojoba oil using Fourier-transform infrared spectroscopy (FTIR) and gas chromatography-mass spectrometry (GC-MS), compare the antimicrobial activity of untreated and IR-treated jojoba oil by determining minimum inhibitory concentrations (MIC) and minimum bactericidal or fungicidal concentrations (MBC/MFC), together with their antibiofilm effects against clinically relevant bacterial and fungal species, assess protein denaturation using a cell-free protein denaturation assay and fibroblast scratch closure potential using a scratch assay in human dermal fibroblasts, determine antiproliferative activity against SK-OV-3 ovarian cancer cells, and investigate associated cellular responses through cell-cycle analysis and expression profiling of *BAX*, *BCL-2*, and *CASP-3*. Through this integrated approach, the study seeks to determine whether IR treatment can meaningfully alter the chemical profile and in vitro biological performance of jojoba oil and thereby provide a scientific basis for its further investigation as a multifunctional natural bioactive material.

## 2. Results and Discussion

### 2.1. Infrared (IR) Irradiation Alters the Molecular and Vibrational Profile of Jojoba Oil: FTIR Spectroscopic Evidence

**Figure 1:**
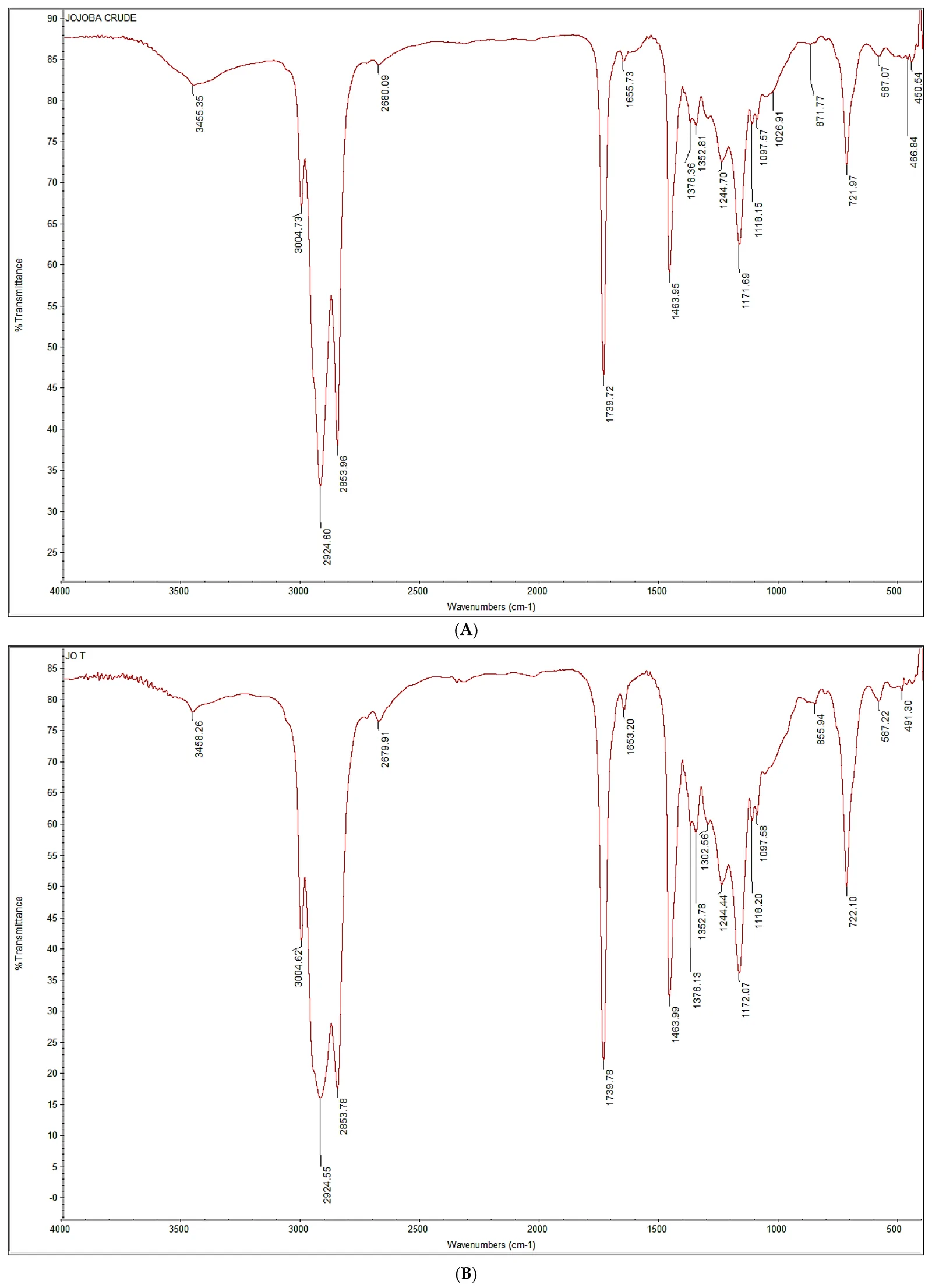
Representative attenuated total reflectance Fourier-transform infrared (FTIR-ATR) spectra of (**A**) crude (untreated) jojoba oil and (**B**) infrared (IR)-treated jojoba oil following 30 min of exposure (780 nm^–1^ mm; 50 mW/cm^2^). Spectra were recorded over the range of 4000–400 cm^−1^ at a resolution of 4 cm^−1^ using 64 scans.

To investigate the physicochemical changes associated with IR irradiation, crude and IR-treated jojoba oils were initially characterized by attenuated total reflectance Fourier-transform infrared (FTIR-ATR) spectroscopy (Figure 1A,B). The spectrum of crude jojoba oil exhibited the characteristic vibrational features of long-chain wax esters, including strong aliphatic C–H stretching bands at 3004, 2924, and 2853 cm^−1^, corresponding predominantly to asymmetric and symmetric CH_2_/CH_3_ vibrations. A sharp ester carbonyl (C=O) stretching band was observed at 1739.7 cm^−1^, together with C–O stretching vibrations at 1244 and 1179 cm^−1^. The characteristic CH_2_ rocking band at 721.9 cm^−1^ was also evident, consistent with extended methylene sequences and the highly ordered long-chain structure of jojoba wax esters [5,25]. In addition, a weak, broad absorption centered at approximately 3455 cm^−1^ was detected in the O–H stretching region and may be attributable to trace moisture or minor hydroxyl-containing constituents, including free fatty alcohols and tocopherols.

Following 30 min of IR treatment, the JO-T spectrum exhibited several distinct changes compared with the crude oil. The thermal control spectrum was essentially indistinguishable from crude oil, with retention of the 1739 cm^−1^ ester carbonyl band and the 721.9 cm^−1^ CH_2_ rocking band and no appearance of the 1633 cm^−1^ band. The most prominent alteration was the marked attenuation of the ester carbonyl band near 1739 cm^−1^, accompanied by the appearance of a moderately intense band at 1633.2 cm^−1^. Absorption within the 1620–1680 cm^−1^ region may be associated with C=C stretching in unsaturated or conjugated structures and, depending on the molecular environment, may also reflect contributions from conjugated carbonyl-containing species [12,26]. Accordingly, the emergence of the 1633.2 cm^−1^ band, together with the reduction in the original ester carbonyl signal, indicates that IR exposure altered the chemical environment of carbonyl- and unsaturation-associated functional groups. These spectral changes may be compatible with processes such as changes in unsaturation, conjugation, oxidative modification, or rearrangement of ester-containing constituents. However, FTIR spectroscopy alone cannot establish specific reactions such as cis–trans isomerization, ester cleavage, decarboxylation, or formation of defined conjugated enone structures.

Such interpretations are nevertheless consistent with previous studies of irradiated lipid systems, in which energy exposure has been associated with radical-mediated reactions involving allylic sites, subsequent molecular rearrangement, conjugation, and oxidative modification [27,28,29]. The pronounced change in the carbonyl region observed here therefore provides evidence that the IR treatment affected the molecular organization and/or relative abundance of functional groups within jojoba oil, although confirmation of the underlying chemical pathways requires complementary compositional analysis.

Additional changes were observed in the O–H stretching region. The broad band centered at approximately 3455 cm^−1^ in crude jojoba oil shifted to approximately 3435 cm^−1^ after IR treatment and became noticeably broader. This change suggests modification of the hydrogen-bonding environment and may reflect an increased contribution from hydroxyl-containing or more polar species. In oxidized lipid matrices, similar spectral behavior has been associated with the formation of hydroperoxides, alcohols, or free fatty acids; however, these individual products cannot be identified unequivocally from the present FTIR spectrum alone. The observed alteration is therefore more appropriately interpreted as evidence of changes in the polarity and intermolecular interactions of the treated oil.

Changes were also evident in the lower-wavenumber fingerprint region. The reduction in intensity of the CH_2_ rocking band at 721.9 cm^−1^ suggests modification of the ordered packing of long aliphatic chains, whereas the appearance of additional bands at 587.2 and 491.3 cm^−1^ provides further evidence that IR irradiation altered the vibrational profile of the oil. These changes could be associated with molecular rearrangement or the generation of chemically distinct minor constituents following treatment. Importantly, the treated oil did not exhibit the extensive spectral complexity that would typically be expected from severe degradation, such as widespread broadening or the appearance of numerous additional carbonyl-related absorptions [30]. Because the matched thermal control was essentially indistinguishable from crude oil and did not reproduce the major IR-associated spectral changes, namely, attenuation of the 1739 cm^−1^ ester carbonyl band, appearance of the 1633 cm^−1^ band, O–H shift/broadening, or reduced CH_2_ rocking, these FTIR changes are attributed to IR treatment beyond the matched bulk-heating profile. Nevertheless, localized microthermal effects or transient hot spots cannot be fully excluded; therefore, the term ‘IR treatment’ is used rather than claiming a purely non-thermal mechanism.

Collectively, the FTIR findings demonstrate that 30 min of IR irradiation produced measurable changes in the molecular and vibrational profile of jojoba oil, particularly in spectral regions associated with ester carbonyl groups, unsaturation, hydroxyl interactions, and long-chain hydrocarbon organization. These results provide the first analytical indication within the present study that IR treatment modified the physicochemical characteristics of the oil and establish the rationale for complementary GC-MS analysis to determine whether these spectroscopic changes were accompanied by detectable alterations in its chemical composition.

For the optimization of IR exposure duration, jojoba oil was exposed to IR for 10–60 min. As shown in Supplementary Table S3, attenuation of the 1739 cm^−1^ ester carbonyl band increased up to 30 min (55% of crude), with no further significant change at 40–60 min (53–54%). The IR-specific 1633 cm^−1^ band appeared weakly at 20 min and was clearly present from 30 min onward. Antimicrobial activity followed the same trend: MIC against *C. albicans* decreased to 7.8 µg/mL at 30 min, and biofilm inhibition against *S. aureus*, *E. faecalis*, *S. typhi*, and *P. aeruginosa* reached maxima at 30 min (96.8%, 91.5%, 88.2%, and 89.5%, respectively). Longer exposures did not improve these values. The 30 min IR-treated oil differed significantly from crude oil and thermal control (*p* < 0.05; Table S1). Therefore, 30 min was selected.

### 2.2. Gas Chromatography-Mass Spectrometry (GC-MS) Profiling Reveals IR-Induced Qualitative and Relative Compositional Alterations in Jojoba Oil

**Figure 2:**
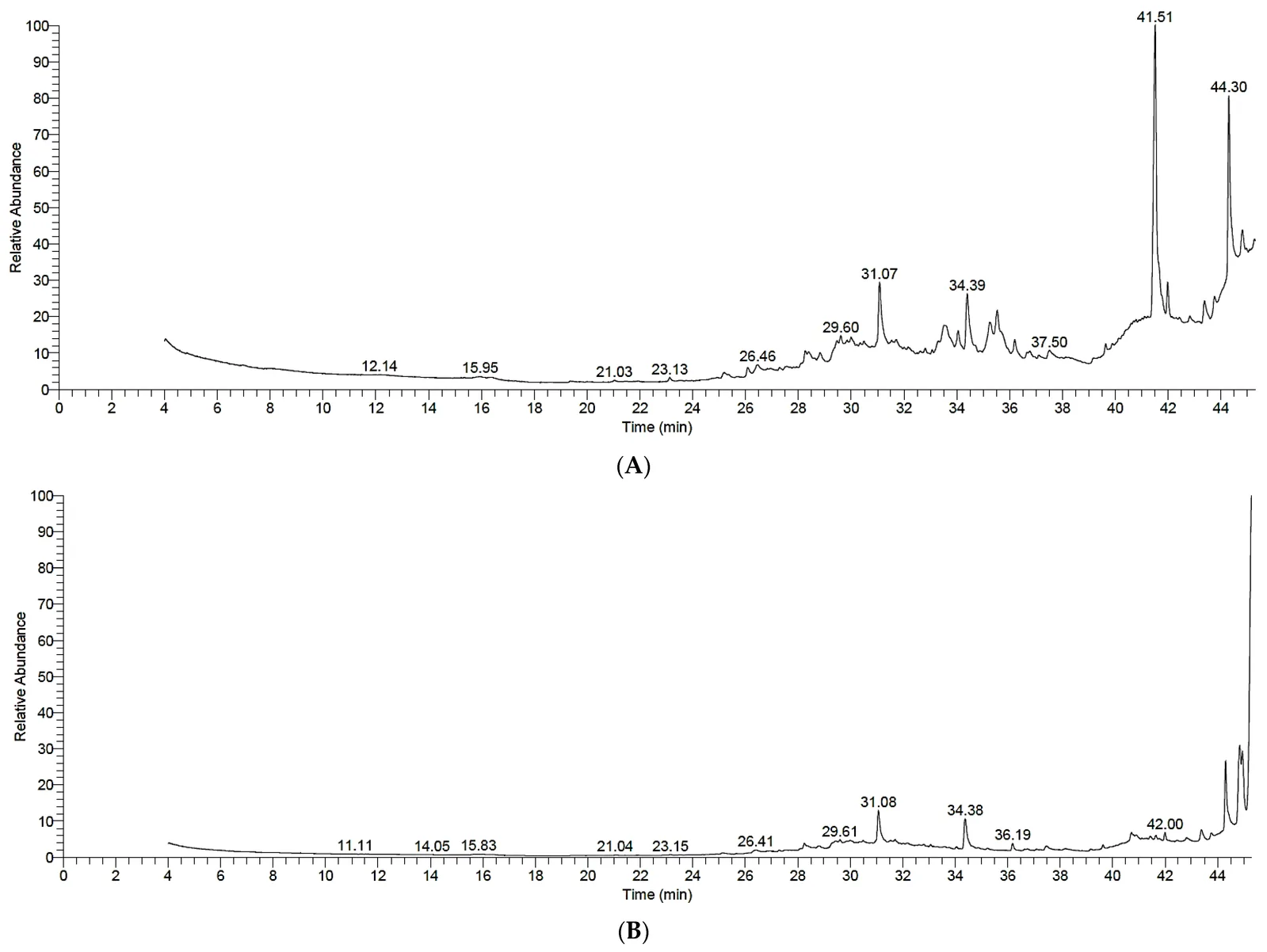
Gas chromatography-mass spectrometry (GC-MS) total ion chromatograms of (**A**) crude (untreated) jojoba oil and (**B**) infrared (IR)-treated jojoba oil following 30 min of exposure at 50 mW/cm^2^. Differences in chromatographic peak patterns and relative peak intensities illustrate the IR-associated changes in the GC-MS-detectable chemical profile of jojoba oil. Compounds were tentatively identified using the NIST 20 library and retention indices. Identification was accepted only when the NIST match score was ≥80, the experimental RI was within ±15 units of the literature RI, and the molecular ion and key fragment ions were consistent with the proposed structure. Compounds failing these criteria were excluded from Table 1 and Table 2 and from all biological interpretation; only accepted tentative identifications are reported. Therefore, relative peak areas are reported without SD, and no statistical inference is drawn from GC-MS comparisons. These data are semi-quantitative and hypothesis-generating.

To complement the FTIR findings and further investigate the compositional changes associated with IR treatment, crude (Table 1 and Figure 2A) and IR-treated (Table 2 and Figure 2B) jojoba oils were analyzed by GC-MS. Comparison of the chromatographic profiles revealed differences in the number and relative abundance of detected constituents, indicating that IR exposure altered the GC-MS-detectable profile of jojoba oil. Because non-detection may reflect detection limits, extraction efficiency, chromatographic behavior, co-elution, or analytical variability, the absence of a peak does not establish complete degradation or elimination. Likewise, the appearance of a new peak does not establish direct formation from a specific precursor or IR-driven reaction. Because compound assignments were based on mass-spectral library matching and no absolute quantification of individual constituents was performed, the identified compounds should be considered tentative, and the reported peak-area percentages represent relative chromatographic abundance rather than absolute concentrations. Accordingly, an increase or decrease in a relative peak area indicates a change in the proportion of the total detected chromatographic area and does not, by itself, demonstrate a corresponding change in the absolute amount of that compound.

The crude jojoba oil profile was dominated by peaks tentatively assigned to major constituents, including β-sitosterol (19.95%), 13-heptadecyn-1-ol (10.66%), 17-octadecynoic acid (6.96%), cis-13-eicosenoic acid (6.34%), and 1-heptatriacotanol (5.80%). Minor constituents tentatively assigned as an epoxy fatty acid ester, friedelan-3-one, and isochiapin B were also detected. Overall, the profile contained several long-chain fatty acids, fatty alcohols, and sterol-related compounds, broadly reflecting the lipid-rich nature of jojoba oil reported previously [9,31]. Nevertheless, the occurrence of individual library-assigned compounds, particularly unusual steroidal or specialized structures, should be interpreted cautiously in the absence of confirmation using authentic standards or complementary structural techniques.

IR treatment for 30 min resulted in a distinctly different chromatographic profile. The thermal control profile was similar to crude oil; 9-hexadecenoic acid, 9-octadecenyl ester (Z,Z), 9-eicosyne, and cis-10-nonadecenoic acid were not detected, and cis-13-eicosenoic acid remained at 6.41%. (Table 2 and Figure 2B, Supplementary Table S1). One of the most prominent changes was the appearance of a major peak at a retention time of 44.85 min, tentatively identified as 9-hexadecenoic acid, 9-octadecenyl ester, (Z,Z)-, which represented 27.45% of the detected peak area and was not detected in the crude oil. This peak was detected only in IR-treated oil under the current GC-MS conditions. This indicates altered detectability or relative composition, but it does not establish that the compound was directly formed by IR treatment or through esterification, transesterification, rearrangement, or another pathway. Detection limits, extraction efficiency, chromatographic behavior, co-elution, and analytical variability cannot be excluded.

Marked changes were also observed among the detected fatty acids and hydrocarbons. The relative peak area assigned to cis-13-eicosenoic acid increased from 6.34% in crude oil to 18.17% after IR treatment, whereas cis-10-nonadecenoic acid, accounting for 4.09%, was detected only in the treated sample. Similarly, a peak tentatively assigned to 9-eicosyne represented 8.97% of the treated-oil chromatographic profile but was not detected in the crude sample. This change reflects its proportion of the total chromatographic area detected and does not establish an increase in its absolute concentration. These findings indicate that IR treatment altered the relative representation of unsaturated lipid-associated constituents. They do not establish decarboxylation, radical-mediated coupling, or any specific reaction mechanism.

Conversely, several constituents detected in the crude oil were no longer detected following IR exposure. A peak tentatively assigned to 9-hexadecenoic acid, octadecenyl ester (Z,Z) was detected only in IR-treated samples, with a relative peak area of 27.45%. These changes in relative peak areas do not establish absolute generation or degradation; they indicate relative enrichment or depletion within the detectable GC-MS profile. Friedelan-3-one and B were likewise absent from the GC-MS profile following irradiation. Their non-detection indicates that they were below the analytical detection threshold under the current GC-MS conditions. This may reflect degradation, transformation, altered volatility, extraction efficiency, chromatographic behavior, co-elution, or analytical variability, but it does not establish complete elimination or conversion. Accordingly, these findings should not be interpreted as definitive evidence of photodegradation, ring opening, or oxidative cleavage without additional structural characterization.

In contrast, β-sitosterol remained a major detectable constituent following treatment, accounting for 21.47% of the chromatographic area compared with 19.95% in crude oil. Because these values represent relative peak areas rather than absolute concentrations, this difference should not be interpreted as a true increase or decrease in the amount of β-sitosterol. The apparent change may also reflect redistribution of the total detected chromatographic area caused by other treatment-associated peaks. Rather, its persistence in both profiles suggests that this sterol-related constituent remained detectable under the applied treatment conditions. The relative peak area of 13-heptadecyn-1-ol decreased from 10.66% to 2.02%, whereas 9-octadecen-1-ol appeared in the treated oil at 9.33%. This indicates a lower proportion of the total detected chromatographic area and does not necessarily indicate a lower absolute amount. Although these differences indicate substantial modification of the alcohol-containing fraction, the present data cannot establish whether the newly detected compound resulted from chain rearrangement, isomerization, or an independent transformation pathway. These comparisons are based on relative peak areas and do not provide quantitative concentration data.

The GC-MS findings are broadly consistent with the FTIR evidence of IR-associated modification of jojoba oil. FTIR demonstrated changes in regions associated with ester carbonyl groups, unsaturation, hydroxyl interactions, and long-chain hydrocarbon organization, whereas GC-MS revealed pronounced differences in the identities and relative peak areas of detectable constituents following treatment. Importantly, the two techniques provide complementary rather than mechanistically definitive information. For example, the detection of a peak tentatively assigned to unsaturated ester-, fatty acid-, alcohol-, and hydrocarbon-related peaks may contribute to the altered vibrational profile observed by FTIR, but the present analyses cannot establish direct precursor-product relationships between individual crude- and treated-oil constituents.

From a biological perspective, these compositional differences are particularly relevant because fatty acids, fatty alcohols, sterol-related compounds, and other lipophilic constituents have previously been associated with antimicrobial, anti-inflammatory, and membrane-interacting activities [7,14,32,33]. Changes in their relative representation could therefore contribute to differences in the biological performance of crude and IR-treated jojoba oil. Nevertheless, direct attribution of a specific biological effect to an individual GC-MS-detected constituent would require isolation of the compound, quantitative confirmation, and activity testing. Consequently, the present GC-MS results are best viewed as a comparative chemical framework for interpreting the biological assays described in the subsequent sections rather than as proof of the molecular mechanisms underlying those effects.

Taken together, the combined FTIR and GC-MS analyses demonstrate that 30 min of IR treatment produced substantial and reproducible changes in the physicochemical and GC-MS-detectable compositional profile of jojoba oil. A notable feature of the present study is the integration of these comparative chemical data with antimicrobial, antibiofilm, anti-inflammatory, fibroblast scratch closure, and antiproliferative activity against SK-OV-3 cell assessments, allowing the biological consequences of IR-associated compositional modification to be investigated within the same experimental framework.

### 2.3. IR-Treated Jojoba Oil Exhibits Enhanced Antimicrobial Activity Against the Tested Pathogens

**Figure 3:**
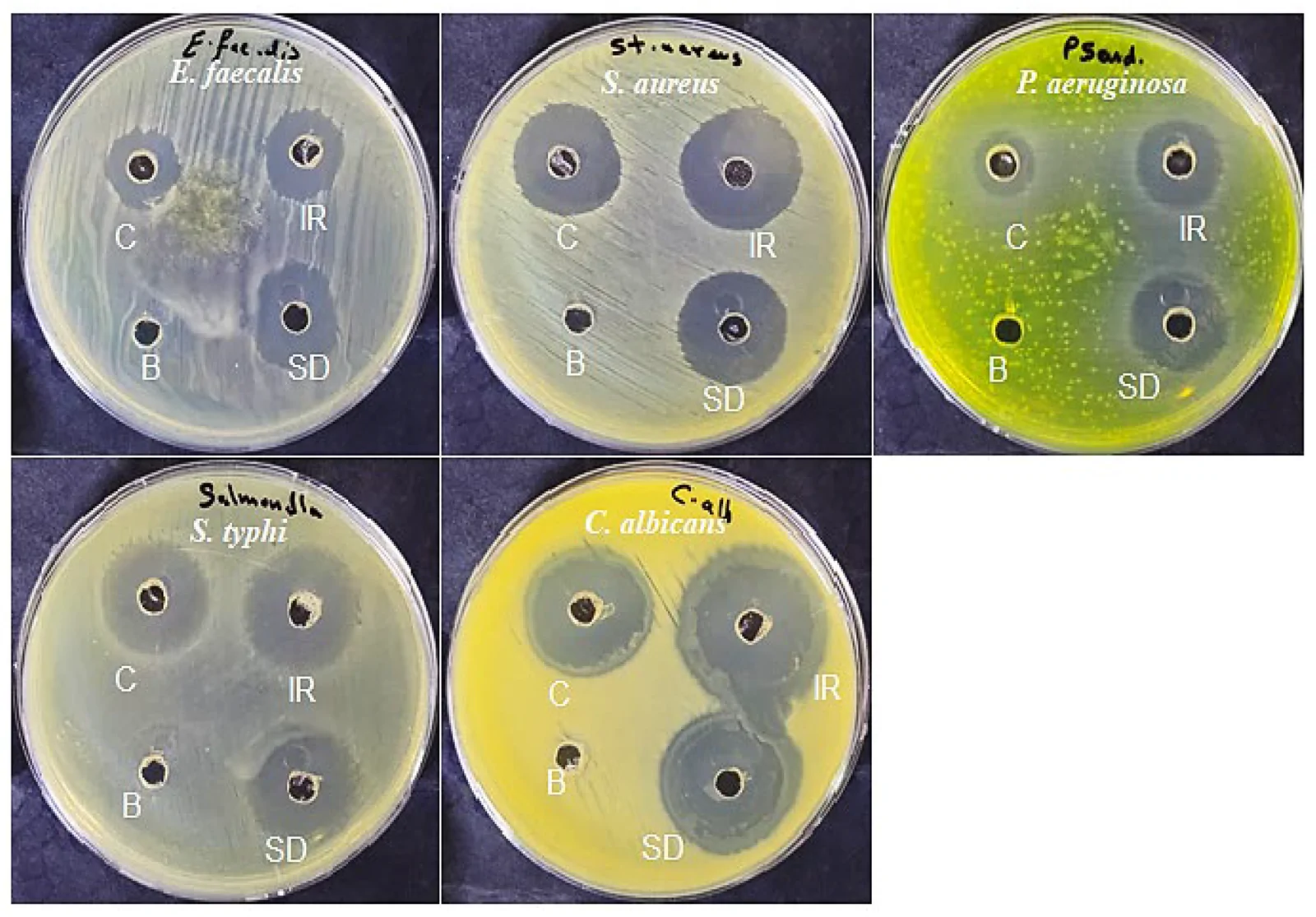
Representative agar well diffusion results for crude (untreated) jojoba oil (C), IR-treated oil (IR), vehicle blank (B), and the positive-control antimicrobial agent (SD) against the tested microorganisms.

The antimicrobial activity of crude and IR-treated jojoba oils was initially evaluated using the agar well diffusion assay (Table 3 and Figure 3). Also, IR treatment reduced the MIC by approximately two- to fourfold across the tested microorganisms. The largest reduction was observed against *P. aeruginosa*, for which the MIC decreased from 62.5 to 15.62 µg/mL (fourfold). Against *C. albicans*, the MIC decreased from 15.62 to 7.8 µg/mL. The overall paired comparison across the five microorganisms was evaluated using the exact two-sided Wilcoxon signed-rank test (*p* = 0.0625), indicating a trend rather than a statistically significant global difference. Per-microorganism comparisons are secondary and exploratory and are reported with effect direction and BH-adjusted *p*-values in Table 4; they are not used to claim global significance.

MBC/MFC values followed the same trend. For *P. aeruginosa*, the MBC decreased from 62.5 to 31.25 µg/mL. For *C. albicans*, the MFC decreased from 31.25 to 15.62 µg/mL. MBC/MIC and MFC/MIC ratios were ≤2 for all IR-treated samples, consistent with bactericidal/fungicidal activity under the assay conditions. However, the agar well diffusion method is influenced by the diffusion and solubility of the test substance within the agar matrix. Because jojoba oil is lipophilic and the antibiotic controls are hydrophilic, the diameters of the oils cannot be directly compared with those of gentamicin or fluconazole. The positive controls produced inhibition zones under the assay conditions, confirming assay performance, but these values do not establish equivalent or superior potency of jojoba oil. The MIC and MBC/MFC data provide the primary concentration-based comparison of antimicrobial activity. These findings are consistent with previous reports describing antimicrobial properties of jojoba oil against a range of microorganisms [2,6], while further demonstrating that IR treatment can substantially modify its activity against the organisms examined in the present study.

The broth microdilution results provided further evidence of the enhanced antimicrobial activity of the treated oil (Table 4). IR treatment reduced the minimum inhibitory concentration (MIC) by approximately two- to fourfold across the tested microorganisms. The most pronounced reduction was observed against *P. aeruginosa*, for which the MIC decreased fourfold, from 62.5 µg/mL for crude jojoba oil to 15.6 µg/mL for the IR-treated oil (*p* = 0.0021). Against *C. albicans*, the MIC decreased from 15.62 to 7.8 µg/mL (*p* = 0.0043). The primary global comparison across the five microorganisms was performed using the exact two-sided Wilcoxon signed-rank test and did not reach the conventional significance threshold (*p* = 0.0625), indicating a trend toward lower MICs after IR treatment. The corresponding one-sided value (*p* = 0.0313) is reported only as a directional sensitivity analysis and is not used as a significance claim. Per-microorganism paired *t*-tests on log_2_-transformed MIC values with Benjamini–Hochberg adjustment are reported in Table 4 as secondary/exploratory analyses; they do not override the non-significant primary global test and should be interpreted as hypothesis-generating. The low variability among three independent experiments (coefficient of variation <5%) further indicated good reproducibility of the antimicrobial measurements.

Minimum bactericidal concentration (MBC) and minimum fungicidal concentration (MFC) measurements further demonstrated that the activity of IR-treated jojoba oil extended beyond growth inhibition (Table 5). MBC/MIC or MFC/MIC ratios were ≤2 for all tested microorganisms, consistent with the commonly applied operational criterion for bactericidal or fungicidal activity under experimental conditions. For *P. aeruginosa*, the MBC decreased from 62.5 µg/mL with crude oil to 31.25 µg/mL following IR treatment, representing a twofold reduction and yielding an MBC/MIC ratio of 2 for the treated oil. Similarly, the MFC against *C. albicans* decreased from 31.25 to 15.62 µg/mL. The overall treatment effect for MBC/MFC values was assessed using the same primary test applied to the MIC data, the exact two-sided Wilcoxon signed-rank test. The paired differences were consistently in the direction of lower values after IR treatment; however, with five paired microorganisms, the smallest attainable two-sided *p*-value is 0.0625, and this comparison is therefore described as a consistent trend (*p* = 0.0625) rather than as a statistically significant overall effect. Per-microorganism MBC/MFC comparisons are reported descriptively as median (range) values in Table 5, with BH-adjusted exploratory *p*-values; because these data are discrete, ordinal, and based on *n* = 3 independent experiments, no ANOVA- or Tukey-based significance claim is made. MBC/MIC and MFC/MIC ratios were ≤ 2 for all IR-treated microorganisms, consistent with the operational criterion for bactericidal/fungicidal activity under the assay conditions.

The enhanced antimicrobial activity observed following IR treatment coincided with the substantial compositional changes identified by FTIR and GC-MS. In particular, the treated oil contained a major peak tentatively assigned to 9-hexadecenoic acid, 9-octadecenyl ester, (Z,Z)-(27.45% relative peak area), together with 9-eicosyne (8.97% relative peak area) and an increased relative chromatographic abundance of cis-13-eicosenoic acid. These values describe relative peak-area distribution, not absolute concentration. Unsaturated lipid constituents have been reported to interact with microbial membranes and may influence membrane organization, permeability, and cellular homeostasis [34]. It is therefore possible that the altered relative distribution of lipid-associated constituents contributed to the enhanced antimicrobial performance of the IR-treated oil. However, because individual compounds were not isolated or independently tested, this remains a possible explanation rather than an established mechanism. No specific compound can be designated as responsible for the observed activity. However, the present experiments do not establish which individual constituent, or combination of constituents, is responsible for the observed effect, and direct structure-activity relationships would require isolation, quantitative characterization, and antimicrobial testing of the candidate compounds.

The improvement observed against *P. aeruginosa* is particularly notable because its outer membrane, efflux systems, and other intrinsic defense mechanisms can limit susceptibility to many antimicrobial compounds, including some plant-derived products [35]. Nevertheless, the agar diffusion results should be interpreted with caution because the lipophilic oil and hydrophilic antibiotics differ substantially in diffusion and solubility within the aqueous agar matrix. The zone diameters therefore do not establish that jojoba oil is as effective as the tested antibiotics. The MIC and MBC/MFC results, which are based on serial dilution in broth, provide a more reliable concentration-dependent comparison under the conditions used here. In the present study, both the inhibition-zone diameter and broth microdilution endpoints showed greater activity of IR-treated jojoba oil against this organism than that of the crude oil. Nevertheless, these findings relate specifically to the strain and experimental conditions examined here and should not be extrapolated to multidrug-resistant clinical isolates without additional testing.

Collectively, the diffusion, MIC, and MBC/MFC findings consistently demonstrate that IR treatment enhanced the in vitro antimicrobial activity of jojoba oil against the tested bacterial and fungal microorganisms. Importantly, the novelty of these findings lies in linking this enhanced antimicrobial phenotype with the IR-associated modification of the oil’s chemical profile within the same experimental framework. Although the specific bioactive constituents and mechanisms responsible remain to be established, the results provide a strong basis for investigating IR treatment as a physical approach for modulating the antimicrobial properties of jojoba oil.

### 2.4. IR-Treated Jojoba Oil Exhibits Enhanced Biofilm Formation Inhibition Activity Against the Tested Bacterial Pathogens

The ability of crude and IR-treated jojoba oils to inhibit biofilm formation was evaluated against the four tested bacterial pathogens using sub-bactericidal concentrations corresponding to 25%, 50%, and 75% of their respective minimum bactericidal concentrations (MBCs) (Table 6 and Supplementary Figure S1). The oil was present during biofilm development; therefore, the assay measures inhibition of biofilm formation, not eradication of pre-existing mature biofilms. Across the concentration range examined, IR-treated jojoba oil generally produced greater inhibition of biofilm biomass accumulation than the crude oil, with the magnitude of inhibition increasing with concentration. These data do not establish whether IR-treated oil can eradicate established biofilms or kill viable cells within them.

At 75% MBC, IR-treated jojoba oil inhibited biofilm formation by more than 92% for all four bacterial species. Thermal control values were similar to crude oil (e.g., *S. aureus* 90.85% vs. 91.32%, *p* > 0.05), while IR-treated oil remained significantly higher (96.80%, *p* < 0.05) (Supplementary Table S1), with the greatest inhibition observed against *S. aureus* (96.80%). Substantial activity was maintained even at 25% MBC. At this lowest tested concentration, biofilm inhibition reached 79.83% for *S. aureus* and 79.04% for *E. faecalis*, compared with 68.01% and 38.08%, respectively, for the crude oil. These findings indicate that the enhancement associated with IR treatment was evident even at concentrations below the bactericidal threshold, suggesting that the observed inhibition of biofilm formation was not simply a consequence of complete bacterial killing.

A particularly pronounced response was observed for *P. aeruginosa*. At 50% MBC, IR-treated jojoba oil inhibited biofilm formation by 91.38%, compared with 82.79% for crude oil (*p* < 0.01). This finding is noteworthy because *P. aeruginosa* readily forms structured biofilms and is frequently associated with persistent infections, including chronic wounds and infections involving medical devices. The substantial inhibition of biofilm formation observed at a sub-MBC concentration therefore adds an important dimension to the antimicrobial activity demonstrated in the preceding experiments.

The enhanced biofilm formation inhibition of the IR-treated oil coincided with the compositional changes identified by FTIR and GC-MS. Among these changes were the detection of a peak tentatively assigned to 9-hexadecenoic acid, 9-octadecenyl ester, (Z,Z), the detection of 9-eicosyne, and changes in the relative abundance of several unsaturated lipid-associated constituents. Lipophilic and unsaturated compounds have previously been associated with effects on bacterial membranes, surface-associated growth, and processes involved in biofilm development [35]. However, the present experiments did not directly assess extracellular polymeric substance disruption, biofilm penetration, quorum-sensing pathways, or the activity of individual GC-MS-detected constituents. Accordingly, these mechanisms remain hypotheses that require targeted experimental confirmation.

Taken together, these findings demonstrate that IR treatment enhanced the ability of jojoba oil to suppress biofilm biomass accumulation during biofilm formation across the tested bacterial species, including concentrations below the corresponding MBCs. Because the assay measured biomass and the oil was added during biofilm development, these results specifically reflect inhibition of biofilm formation and do not demonstrate eradication of mature biofilms or killing of viable cells within established biofilms. The integration of biofilm formation inhibition testing with comparative chemical profiling is an important aspect of the present study, as it shows that the IR-associated modification of jojoba oil was accompanied not only by improved activity against planktonic microorganisms but also by stronger inhibition of surface-associated bacterial growth. This combined antimicrobial and antibiofilm response supports further investigation of IR-treated jojoba oil as a modified natural product for applications in which microbial persistence and biofilm formation are important considerations.

### 2.5. IR-Treated Jojoba Oil Exhibits Enhanced Concentration-Dependent Inhibition of Protein Denaturation

**Figure 4:**
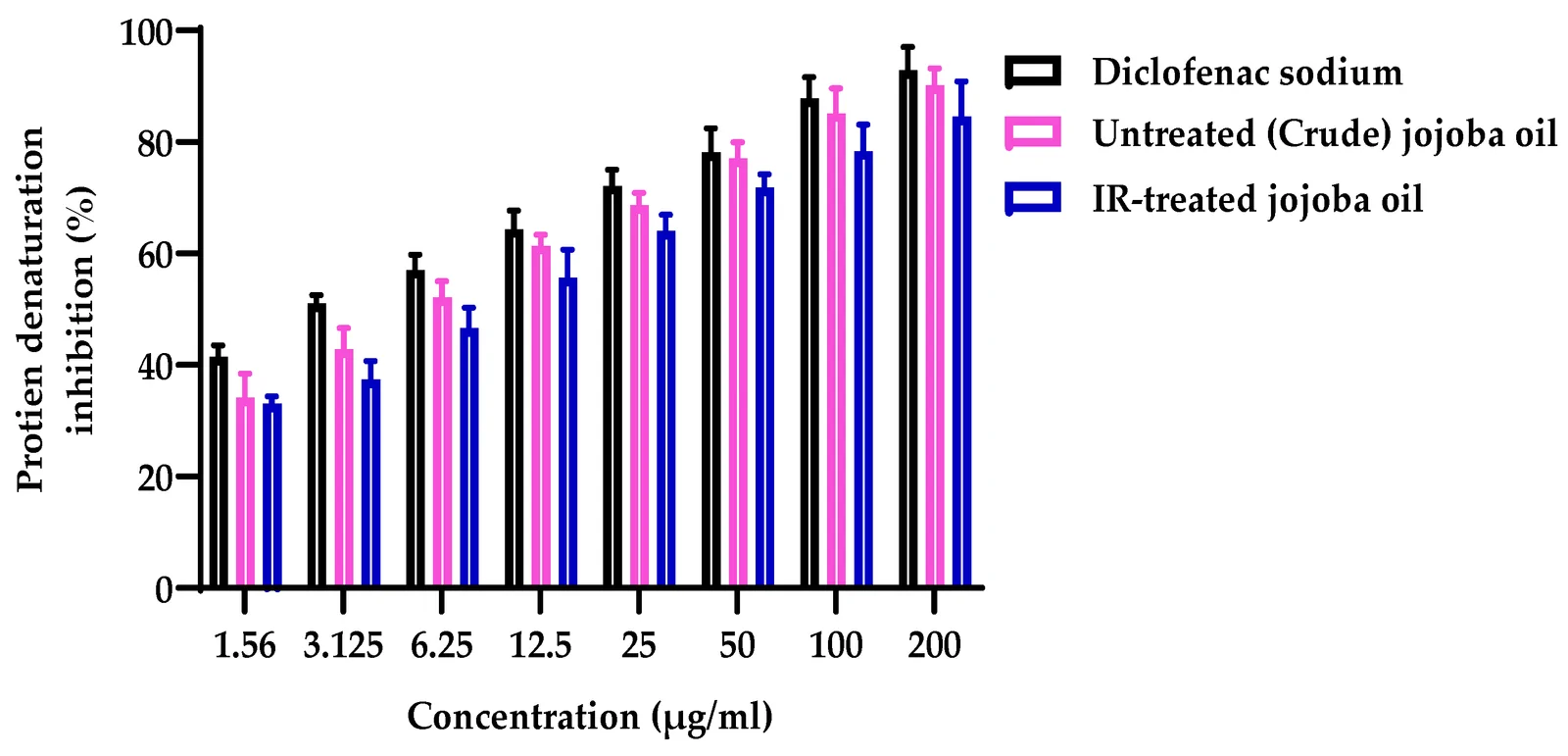
Inhibition of bovine serum albumin (BSA) denaturation by diclofenac sodium (Dicl. St.; positive control), infrared (IR)-treated jojoba oil, and crude jojoba oil. Data are presented as mean ± SD from three independent experiments, each performed in triplicate, with technical replicates averaged within each independent experiment. Concentration–response curves were fitted using nonlinear regression, and IC_50_ values were compared using the extra sum-of-squares F-test. Concentration-specific comparisons were performed using two-way analysis of variance (ANOVA), followed by Sidak’s multiple-comparison test. The IC_50_ values were 4.43 ± 0.02 µg/mL for IR-treated jojoba oil, 6.52 ± 0.10 µg/mL for crude jojoba oil, and 2.75 ± 0.02 µg/mL for diclofenac sodium. Adjusted *p* < 0.05 was considered statistically significant. This cell-free assay measures protein denaturation and does not establish anti-inflammatory efficacy. At 200 µg/mL, inhibition reached 92.3% for IR-treated jojoba oil, 89.0% for crude jojoba oil, and 95.8% for diclofenac sodium.

The anti-denaturation activity of crude and IR-treated jojoba oils was evaluated using the bovine serum albumin (BSA) denaturation assay, an established in vitro screening method commonly used to assess the potential anti-inflammatory properties of bioactive substances [36]. Because protein denaturation can contribute to inflammatory processes, inhibition of heat-induced protein denaturation provides a useful preliminary measure of protein-stabilizing activity. As shown in Figure 4, both crude and IR-treated jojoba oils inhibited protein denaturation in a concentration-dependent manner. Diclofenac sodium, used as the positive control, exhibited an IC_50_ of 2.75 ± 0.015 µg/mL.

IR-treated jojoba oil showed greater inhibitory activity than the crude oil, with an IC_50_ of 4.43 ± 0.015 μg/mL compared with 6.52 ± 0.102 μg/mL for crude jojoba oil (*p* < 0.01). The thermal control IC_50_ was 6.48 ± 0.09 μg/mL, not significantly different from crude oil (*p* > 0.05) (Supplementary Table S1). At the highest tested concentration of 200 µg/mL, IR-treated oil inhibited protein denaturation by 92.3%, compared with 95.8% for diclofenac sodium. Greater inhibition by the IR-treated oil was also observed across the lower concentration range. At 25 µg/mL, inhibition increased from 66.1% with crude oil to 70.2% following IR treatment (*p* < 0.05), while at 12.5 µg/mL the corresponding values were 59.2% and 62.8%, respectively (*p* < 0.05). Collectively, these findings indicate that IR treatment enhanced the ability of jojoba oil to inhibit heat-induced protein denaturation under the cell-free conditions used here. These data support inhibition of protein denaturation only and do not demonstrate an anti-inflammatory effect at the cellular or organismal level.

The improvement in protein denaturation inhibition coincided with the marked compositional changes identified by FTIR and GC-MS. In particular, IR-treated jojoba oil retained a substantial relative abundance of the sterol tentatively assigned as β-sitosterol (21.47%), while also exhibiting changes in several unsaturated lipid-associated constituents. Phytosterols, including β-sitosterol, have been associated with anti-inflammatory activity in previous studies [37], whereas long-chain fatty alcohols, tocopherols, and other lipophilic constituents of jojoba oil have also been proposed to contribute to its biological properties [7]. It is therefore plausible that changes in the relative distribution of such constituents coincide with the greater anti-denaturation activity observed after IR treatment. However, the present assay does not identify the compound or combination of compounds responsible for this effect, and the contribution of individual constituents would require targeted isolation and activity testing. The relationship remains a possible explanation rather than an established mechanism.

Importantly, the high level of inhibition detected only in IR-treated jojoba oil at 200 µg/mL approached that of diclofenac under the assay conditions. However, this comparison should not be interpreted as evidence of equivalent pharmacological efficacy. Diclofenac is a clinically established nonsteroidal anti-inflammatory drug with defined molecular targets, whereas the protein denaturation represents only an in vitro screening model.

Taken together, these results demonstrate that IR treatment significantly enhanced the concentration-dependent inhibition of protein denaturation of jojoba oil. When considered alongside the antimicrobial and biofilm formation inhibition findings, this effect adds to the multifunctional in vitro biological profile of the treated oil. Nevertheless, confirmation of anti-inflammatory potential and elucidation of the underlying mechanisms will require complementary cellular and in vivo models involving established inflammatory mediators and signaling pathways. The present BSA assay alone cannot establish anti-inflammatory efficacy.

### 2.6. IR-Treated Jojoba Oil Exhibits Enhanced In Vitro Antiproliferative/Cytotoxic Activity Against SK-OV-3 Ovarian Cancer Cells

**Figure 5:**
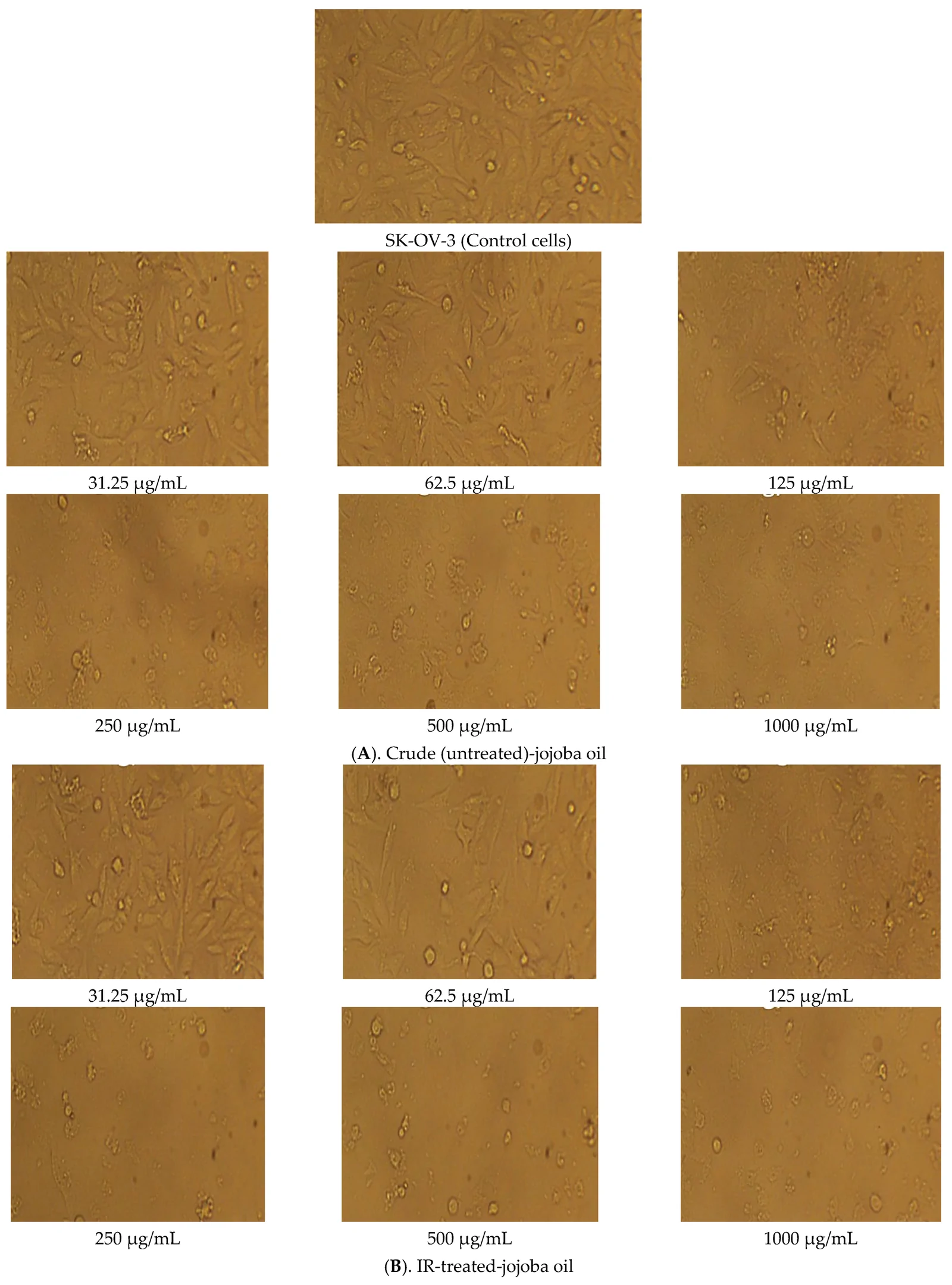

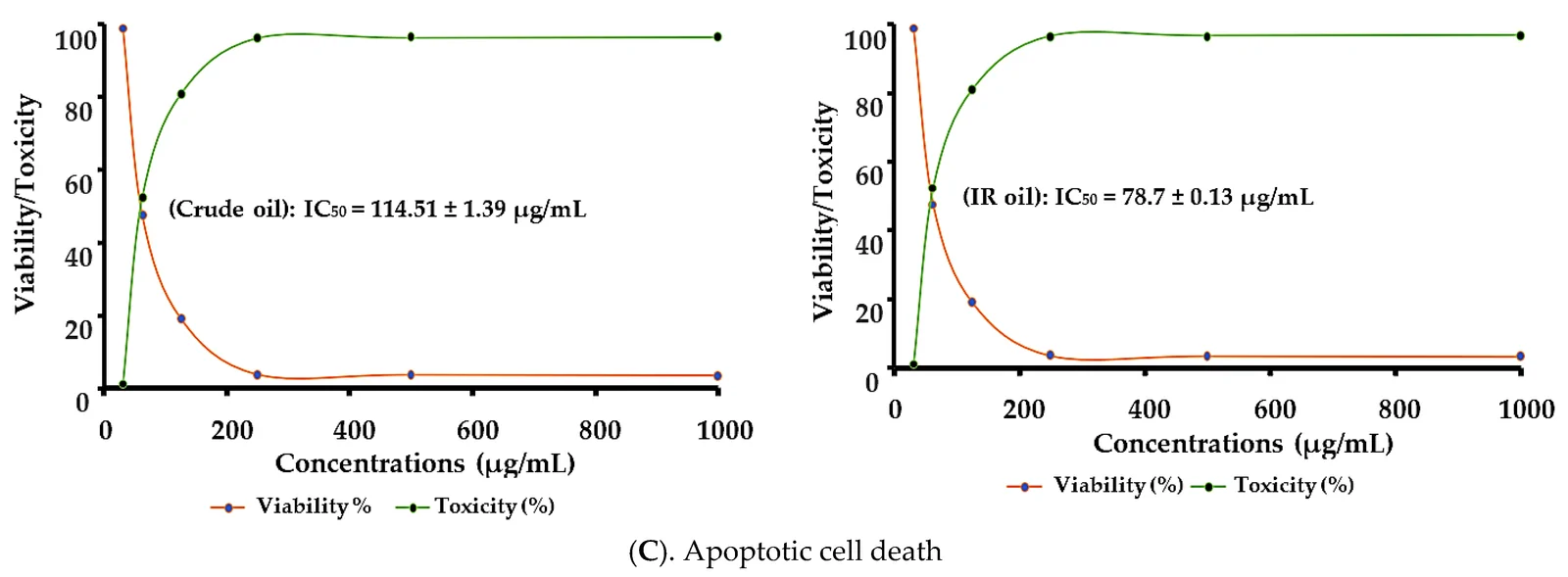
Concentration-response curves and representative morphology are shown. IC_50_ values were 114.5 µg/mL for crude oil and 78.7 µg/mL for IR-treated oil. (**A**,**B**). Representative microscopic images of SK-OV-3 cells after 24 h of exposure to the respective IC_50_ concentrations of crude jojoba oil (114.5 µg/mL) and IR-treated jojoba oil (78.7 µg/mL). Vehicle-treated control cells maintained an adherent, polygonal morphology with an intact monolayer. Cells exposed to crude jojoba oil exhibited morphological alterations characterized by cell shrinkage, rounding, reduced adherence, and detachment. More pronounced morphological changes were observed following treatment with IR-treated jojoba oil, including extensive cell rounding, shrinkage, detachment, and disruption of monolayer integrity. These changes indicate treatment-associated cellular injury and are compatible with, but do not independently establish, apoptotic cell death. Scale bar = 50 µm. (**C**) Concentration–response curves showing the effects of crude and IR-treated jojoba oil on SK-OV-3 cell viability after 24 h of exposure, as determined by the MTT assay. The corresponding IC_50_ values were 114.5 µg/mL for crude (untreated) jojoba oil and 78.7 µg/mL for IR-treated jojoba oil.

The antiproliferative effects of crude and IR-treated jojoba oils were evaluated against SK-OV-3 human ovarian cancer cells using the MTT assay (Table 7 and Figure 5). Both oil preparations produced a concentration-dependent reduction in cell viability; however, IR-treated jojoba oil consistently exhibited greater activity. The IC_50_ of the IR-treated oil was 78.70 ± 0.13 µg/mL, compared with 114.51 ± 1.39 µg/mL for crude jojoba oil, corresponding to an approximately 1.46-fold lower IC_50_ following IR treatment (*p* < 0.01, unpaired *t*-test). The thermal control IC_50_ was 112.84 ± 1.25 μg/mL, similar to crude oil (*p* > 0.05) (Supplementary Table S1). These findings indicate that modification of jojoba oil by IR exposure significantly enhanced its ability to reduce SK-OV-3 cell viability under the experimental conditions. Because no normal ovarian or other nonmalignant cell line was tested in parallel, these data do not establish cancer-cell selectivity, preferential toxicity, or a therapeutic window.

The difference between the two oils was particularly evident at 125 µg/mL, where cell viability decreased to 42.05% following treatment with crude jojoba oil and to 19.06% following treatment with IR-treated oil, corresponding to reductions in viability of 57.95% and 80.94%, respectively (*p* < 0.001). At higher concentrations (250–1000 µg/mL), IR-treated jojoba oil reduced cell viability by more than 96%, whereas crude oil produced reductions of approximately 93–96%. Thus, although both oils exhibited substantial activity at higher concentrations, the lower IC_50_ and greater reduction in viability at intermediate concentrations demonstrate that IR treatment enhanced the antiproliferative response of jojoba oil.

Microscopic examination of SK-OV-3 cells provided complementary morphological evidence of treatment-associated cellular injury (Figure 5). Untreated control cells maintained a confluent monolayer with the characteristic adherent morphology of SK-OV-3 cells. Exposure to crude jojoba oil at its IC_50_ resulted in visible morphological alterations, including cell rounding, reduced adherence, and an increased number of detached cells. More pronounced changes were observed following exposure to IR-treated jojoba oil at its lower IC_50_ of 78.70 µg/mL, including extensive cell shrinkage, rounding, detachment, and disruption of monolayer integrity. These morphological changes are compatible with substantial cellular stress and loss of viability. Although some of these features can accompany apoptotic cell death, morphology alone is insufficient to establish apoptosis or distinguish it from other modes of cell death. This possibility was therefore investigated further using cell-cycle analysis and apoptosis-associated gene-expression measurements.

The enhanced antiproliferative activity of IR-treated jojoba oil occurred in parallel with the substantial chemical differences identified by FTIR and GC-MS. The treated oil exhibited changes in several lipid-associated constituents, including the detection of a peak tentatively assigned to 9-hexadecenoic acid, 9-octadecenyl ester, (Z,Z)-(27.45% relative peak area), and an increase in the relative chromatographic abundance of cis-13-eicosenoic acid (18.17% relative peak area). These observations indicate altered relative composition, not absolute accumulation of these compounds. Unsaturated fatty acids and related lipids have been reported to influence cancer-cell membrane properties, cellular metabolism, oxidative homeostasis, and signaling pathways associated with proliferation and cell death [38,39,40]. Changes in the relative distribution of these or other constituents could therefore contribute to the greater biological activity of the IR-treated oil. However, the present results do not establish that any individual GC-MS-detected compound is directly responsible for the observed antiproliferative effect. Such a relationship would require isolation, quantitative confirmation, and independent activity testing of the individual constituents.

It is also important to distinguish enhanced activity against SK-OV-3 cells from selective antiproliferative activity against SK-OV-3 cells. Because no nonmalignant ovarian or other appropriate normal cell model was evaluated in parallel under identical assay conditions, cancer-cell selectivity and a therapeutic window cannot be established from these data. The HDFa scratch assay used a different endpoint, concentration, and assay format and therefore cannot serve as a selectivity control. Accordingly, all anticancer-related findings are limited to in vitro antiproliferative/cytotoxic effects in the SK-OV-3 cell line and should not be extrapolated to selective anticancer activity or clinical efficacy. Previous findings concerning the cytotoxicity of jojoba-derived preparations toward nonmalignant cells may provide useful context, but they cannot substitute for a direct selectivity assessment under the same experimental conditions.

Overall, the MTT and morphological findings indicate that IR treatment enhanced the in vitro antiproliferative/cytotoxic activity of jojoba oil against SK-OV-3 ovarian cancer cells under the tested conditions. This observation extends the in vitro effects associated with IR treatment beyond the antimicrobial, biofilm formation inhibition, and protein denaturation inhibition described above. However, these data are limited to a single cancer cell line; no normal-cell comparator was included, and no in vivo validation was performed. Therefore, these findings do not establish jojoba oil as an anticancer therapeutic or a therapeutic candidate. They warrant further investigation, including normal-cell selectivity testing, protein-level apoptosis assays, and in vivo models, before any antiproliferative activity against SK-OV-3 cells can be evaluated.

### 2.7. IR-Treated Jojoba Oil Enhances Fibroblast Scratch Closure in Human Dermal Fibroblasts

**Figure 6:**
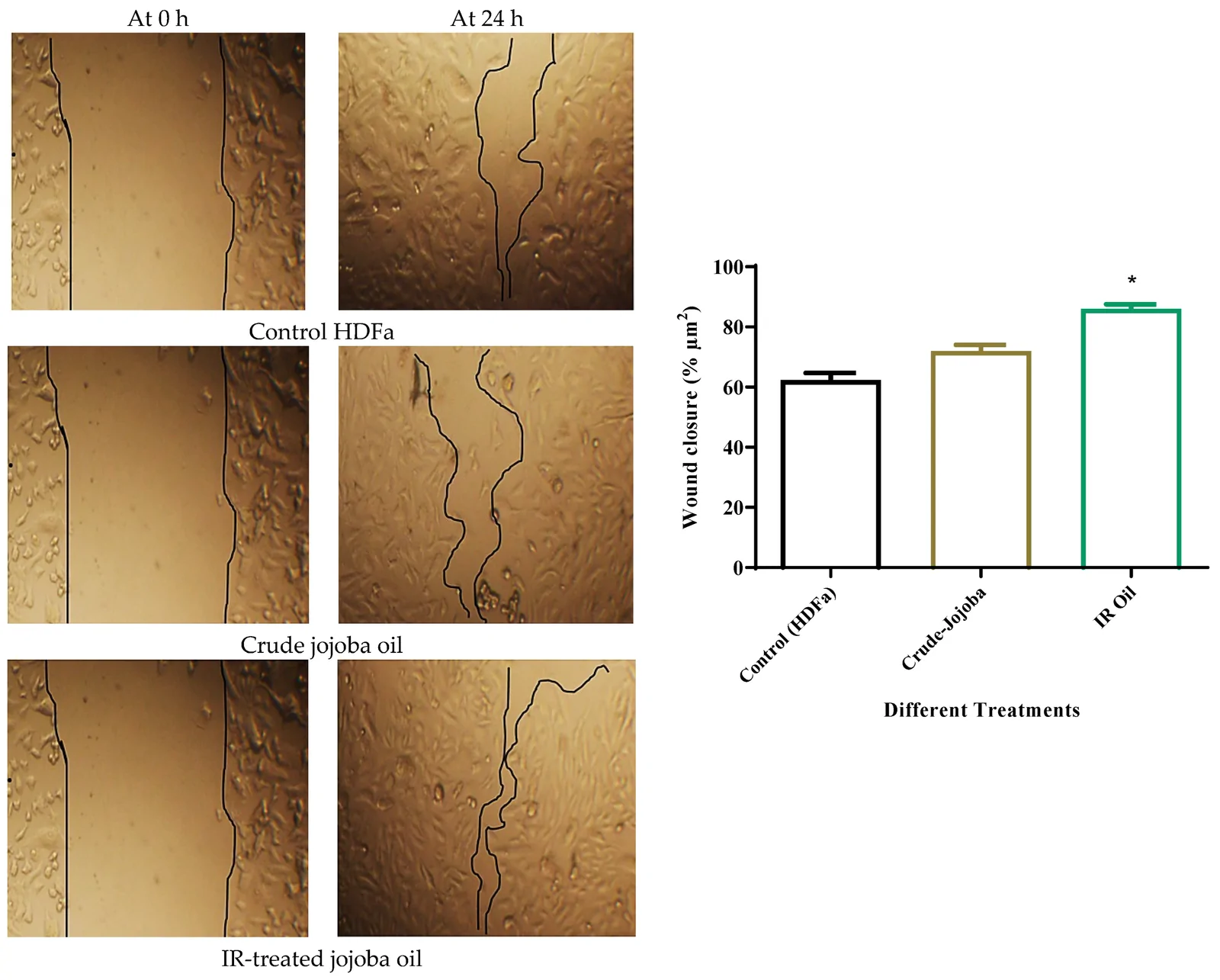
Effect of crude (untreated) and infrared (IR)-treated jojoba oil on in vitro scratch closure in human dermal fibroblasts (HDFa). (**Left panel**): Representative phase-contrast microscopy images of the scratch area at 0 h (left column) and 24 h (right column) in untreated control cells (top row), cells treated with crude jojoba oil (middle row; 125 µg/mL), and cells treated with IR-treated jojoba oil (bottom row; 125 µg/mL). Dashed lines indicate the boundaries of the cell-free area. Scale bar = 200 µm. After 24 h, cells treated with IR-treated jojoba oil exhibited the smallest residual scratch area, consistent with the quantitative assessment of scratch closure. (**Right panel**): Quantitative analysis of wound closure (µm^2^). Data are presented as mean ± SD from three independent experiments, each comprising two replicate scratches (six replicate scratches in total); technical replicates were averaged within each independent experiment and were not treated as independent observations. Statistical comparisons were performed using one-way ANOVA, followed by Tukey’s honestly significant difference (HSD) post hoc test. * Adjusted *p* < 0.05 was considered statistically significant.

Given the established use of jojoba oil in dermatological formulations and its emollient properties, the effects of crude and IR-treated jojoba oils on in vitro fibroblast scratch closure were evaluated using a scratch assay in human dermal fibroblasts (HDFa). A concentration of 125 µg/mL was selected on the basis of preliminary cytotoxicity testing, which showed >80% fibroblast viability at this concentration. As shown in Table 8 and Figure 6, IR-treated jojoba oil significantly enhanced scratch closure compared with both crude oil and untreated control after 24 h of exposure.

Untreated fibroblasts exhibited 67.02% closure after 24 h, corresponding to a residual scratch area of 355.90 ± 103.8 µm^2^. Cells treated with crude jojoba oil showed 76.08% closure, which was not significantly different from the untreated control (*p* = 0.1247). In contrast, fibroblasts exposed to IR-treated jojoba oil exhibited 84.12% closure, with a residual scratch area of 171.37 ± 218.8 µm^2^ and a calculated migration rate of 18.92 µm/h. This represented a 10.6% greater closure response than crude jojoba oil and a 25.5% increase relative to the untreated control (*p* < 0.05).

The greater scratch closure detected only in IR-treated jojoba oil occurred alongside the substantial compositional differences identified by FTIR and GC-MS. In particular, IR treatment was associated with changes in several sterol- and lipid-related constituents, including retention of the compound tentatively assigned as β-sitosterol (21.47%), detection of a peak tentatively assigned to a major unsaturated fatty acid ester (27.45%), and an increase in the relative chromatographic abundance of cis-13-eicosenoic acid (18.17%). Phytosterols and unsaturated lipid constituents have previously been associated with cellular processes relevant to tissue repair, including fibroblast or keratinocyte responses and extracellular matrix regulation [41]. These compositional changes may therefore contribute to the enhanced scratch-closure response observed with the treated oil. However, the present experiments do not establish a direct causal relationship between any individual GC-MS-detected constituent and fibroblast behavior. The observed difference could reflect the combined influence of multiple compositional changes rather than the loss or enrichment of a single compound. Likewise, although pathways such as PI3K/Akt and MAPK are well-established regulators of fibroblast proliferation and migration, their involvement in the response to IR-treated jojoba oil was not investigated and therefore remains a potential mechanism for future study rather than a conclusion of the present work.

Interpretation of the scratch assay should also consider that closure of the cell-free area may result from both cellular migration and proliferation [42]. Consequently, the increased closure observed with IR-treated jojoba oil cannot be attributed specifically to enhanced migration without experiments designed to control cell proliferation. Nevertheless, the significantly greater scratch closure in the IR-treated group compared with both crude oil and untreated cells—although crude oil alone did not differ significantly from control—provides evidence that IR treatment enhanced the capacity of jojoba oil to support fibroblast-mediated gap closure in vitro.

When considered together with the antimicrobial, inhibition of biofilm formation, and protein denaturation findings, the fibroblast response further expands the multifunctional biological profile associated with IR-treated jojoba oil. The combination of enhanced antimicrobial activity and improved fibroblast scratch closure is particularly relevant to future investigation of the treated oil in fibroblast scratch closure-related applications, where control of microbial growth and support of tissue repair are both desirable. However, confirmation of true fibroblast scratch closure efficacy will require more physiologically representative models, including assessment of cell migration and proliferation independently, extracellular matrix deposition, inflammatory responses, and ultimately appropriate in vivo fibroblast scratch closure studies.

### 2.8. IR-Treated Jojoba Oil Promotes Pronounced G0/G1 Accumulation in SK-OV-3 Ovarian Cancer Cells

**Figure 7:**
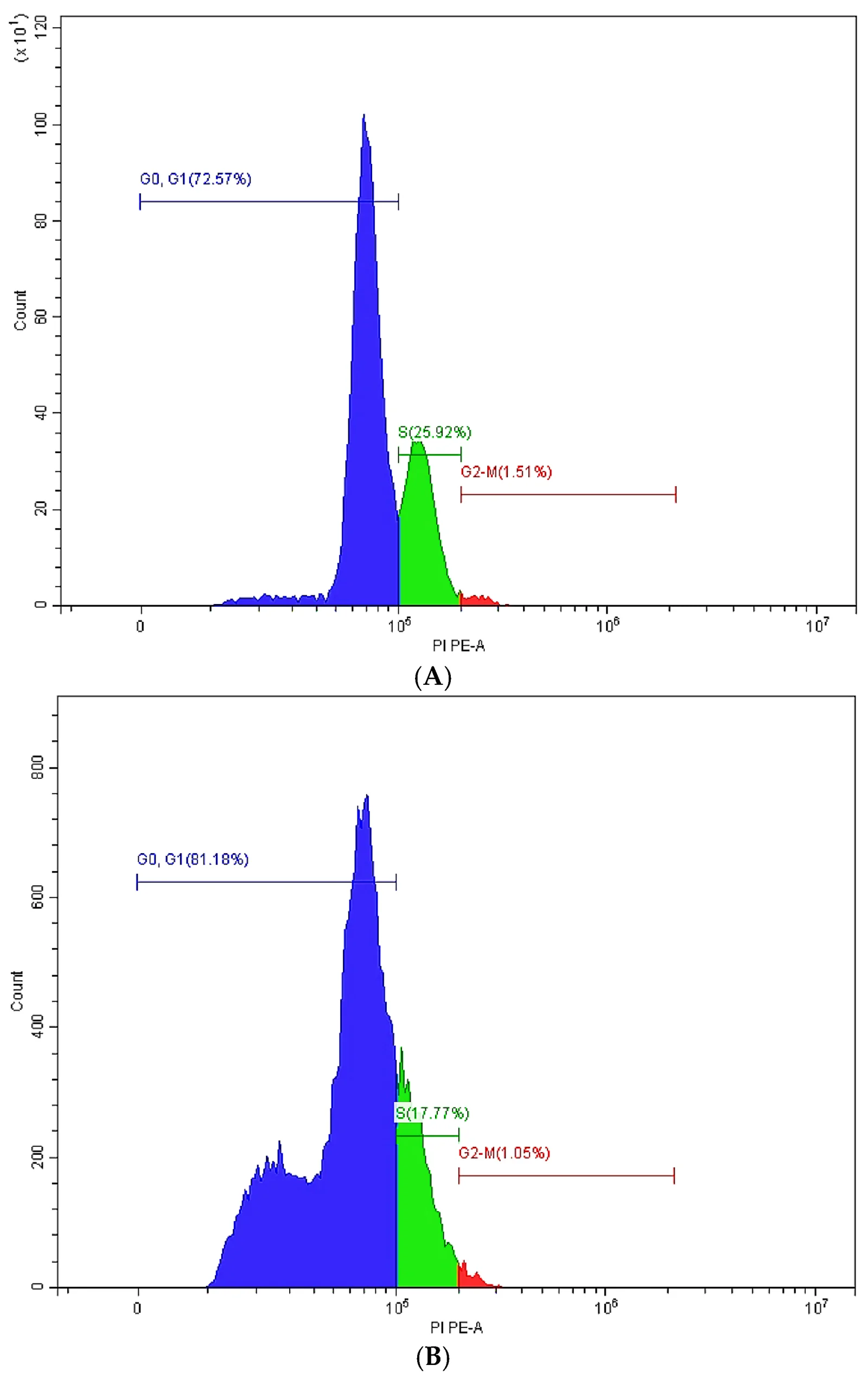

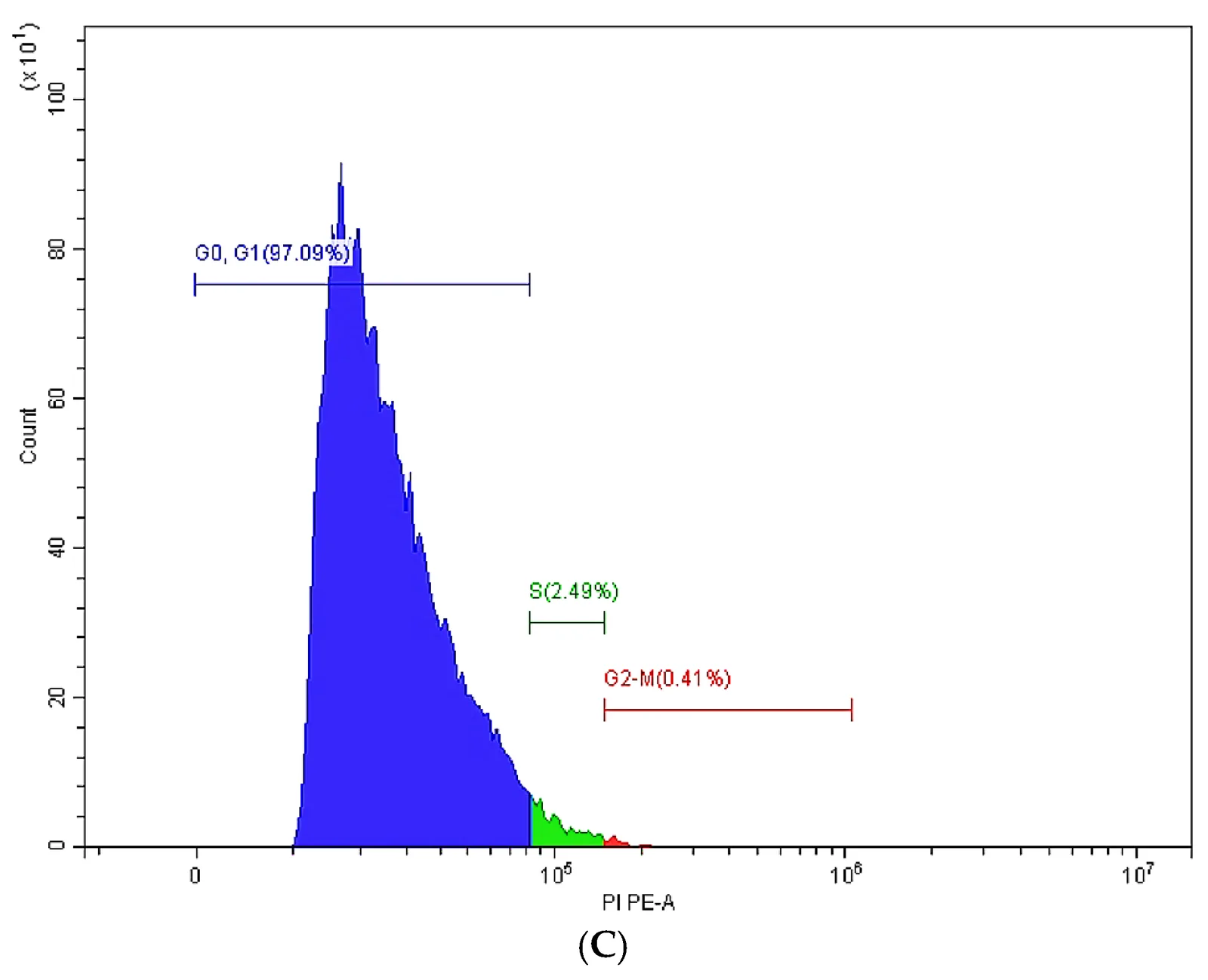
Representative flow cytometry histograms showing cell-cycle distribution in SK-OV-3 human ovarian cancer cells after 24 h of treatment. DNA content was assessed by propidium iodide staining in (**A**) untreated control cells, (**B**) cells treated with crude jojoba oil at its IC_50_ concentration (114.5 µg/mL), and (**C**) cells treated with infrared (IR)-treated jojoba oil at its IC_50_ concentration (78.7 µg/mL). The first major peak represents the G0/G1 population, the intermediate region corresponds to the S phase, and the second peak represents the G2/M population. IR-treated jojoba oil produced a pronounced accumulation of cells in G0/G1 accompanied by marked reductions in the S- and G2/M-phase populations, whereas crude jojoba oil produced a more moderate shift toward G0/G1 with a larger proportion of cells remaining in the S phase.

To further investigate the cellular basis of the antiproliferative activity of jojoba oil, cell-cycle distribution was analyzed in SK-OV-3 ovarian cancer cells after 24 h of exposure to the respective IC_50_ concentrations of crude and infrared (IR)-treated oils (Table 9 and Figure 7). Untreated control cells exhibited a typical proliferative distribution, with 72.5% of the cell population in G0/G1, 25.9% in the S phase, and 1.5% in G2/M.

Exposure to crude jojoba oil resulted in a significant redistribution of cells toward the G0/G1 phase. The G0/G1 population increased from 72.5% in untreated cells to 81.1% following crude-oil treatment (*p* < 0.05), accompanied by a reduction in the S-phase population from 25.9% to 17.7% (*p* < 0.05). The proportion of cells in G2/M decreased slightly to 1.05%, although this difference was not statistically significant. This shift toward G0/G1, together with the reduction in S-phase cells, is consistent with partial suppression of G1-to-S-phase progression and provides a potential cellular explanation for the reduced viability observed in the MTT assay.

A substantially greater alteration in cell-cycle distribution was observed following treatment with IR-treated jojoba oil. The proportion of cells in G0/G1 increased to 97.9%, significantly higher than that observed in both untreated and crude-oil-treated cells (*p* < 0.01). Thermal control showed 81.8% G0/G1, similar to crude oil (81.1%, *p* > 0.05), whereas IR-treated oil showed 97.9% (*p* < 0.01) (Supplementary Table S1). This accumulation was accompanied by a pronounced reduction in the S-phase population to 2.4% (*p* < 0.01) and a decrease in the G2/M population to 0.4% (*p* < 0.05). Comparisons between the two oil treatments likewise demonstrated significant differences in G0/G1 and S-phase distributions (*p* < 0.01), with a smaller but significant difference in G2/M (*p* < 0.05). Collectively, these findings demonstrate that IR-treated jojoba oil produced a markedly stronger redistribution of SK-OV-3 cells toward G0/G1 and a corresponding depletion of cells undergoing DNA synthesis.

The pronounced G0/G1 accumulation observed following IR-treated jojoba oil exposure is consistent with inhibition of cell-cycle progression before entry into the S phase. However, DNA-content analysis does not distinguish quiescent G0 cells from G1-phase cells and, by itself, cannot identify the molecular regulators responsible for this redistribution. Accordingly, the present findings are more appropriately interpreted as evidence of G0/G1 accumulation consistent with cell-cycle arrest rather than direct demonstration of a specific G1 checkpoint mechanism.

The stronger cell-cycle response to IR-treated oil occurred alongside the substantial compositional alterations identified by FTIR and GC-MS, including the appearance or increased relative abundance of several unsaturated lipid-associated constituents. Bioactive lipids and related natural products have been reported to modulate regulators of G1-to-S-phase progression, including cyclins, cyclin-dependent kinases, and their endogenous inhibitors [35]. Such pathways therefore represent plausible mechanisms through which the altered chemical profile of IR-treated jojoba oil could influence cell-cycle progression. However, p21, p27, cyclin D, CDK4/6, and related checkpoint proteins were not measured, and their involvement cannot be established from the current data. The relationship between chemical composition and cell-cycle effects remains a possible explanation, not a demonstrated mechanism.

Importantly, the pronounced depletion of the S-phase population complements the lower IC_50_ observed for IR-treated jojoba oil in the viability assay, providing additional evidence that IR treatment altered the cellular response of SK-OV-3 cells. Together, these findings indicate that the enhanced antiproliferative effect of IR-treated jojoba oil is associated with marked disruption of normal cell-cycle progression, characterized predominantly by accumulation in G0/G1 and reduced entry into the S phase. Whether this cell-cycle disturbance is accompanied by activation of programmed cell-death pathways was subsequently investigated through analysis of apoptosis-associated gene expression.

### 2.9. IR-Treated Jojoba Oil Modulates Apoptosis-Related Gene Expression in SK-OV-3 Ovarian Cancer Cells

**Figure 8:**
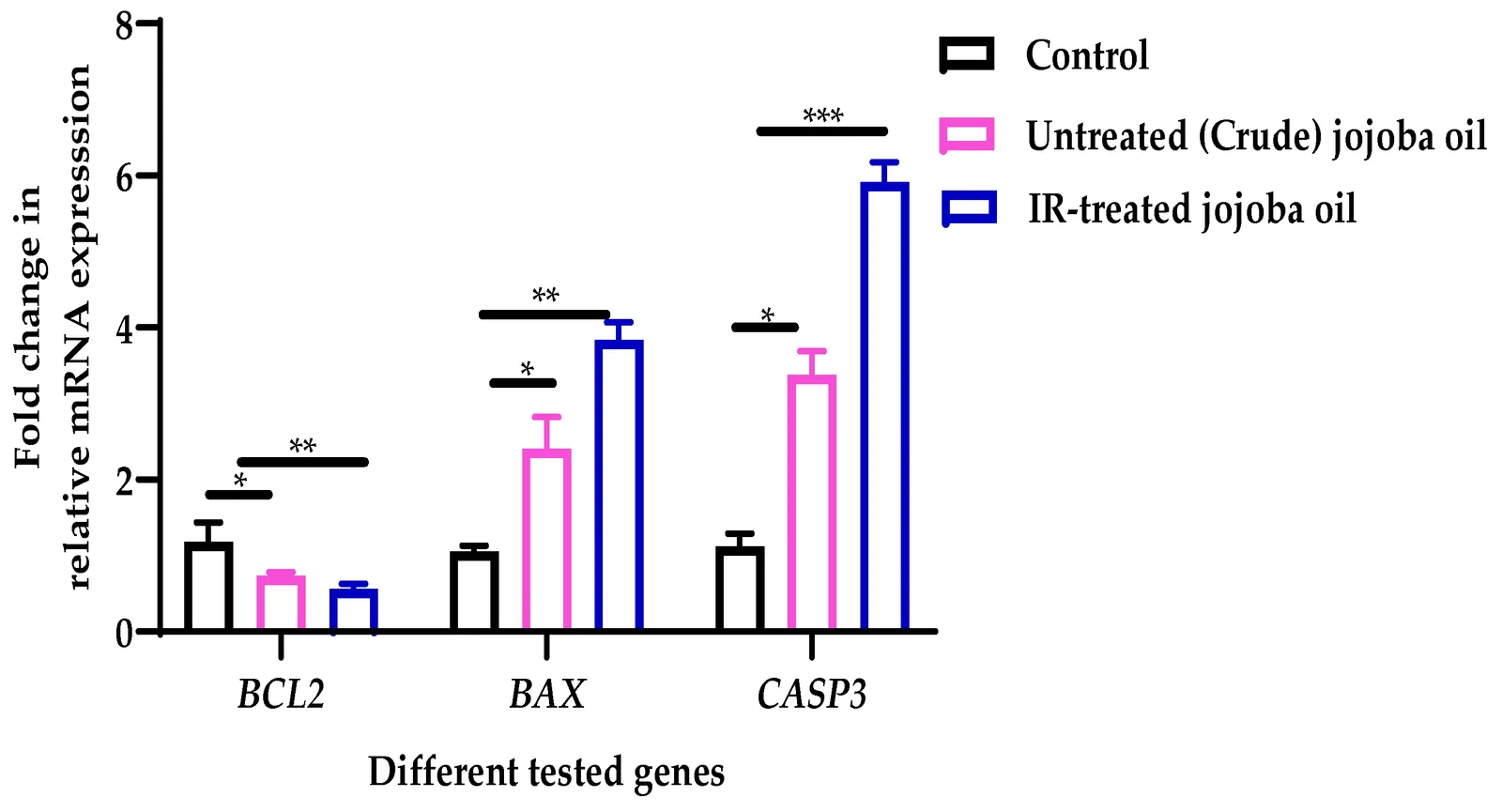
Quantitative reverse-transcription polymerase chain reaction (qRT-PCR) analysis of apoptosis-related gene expression in SK-OV-3 human ovarian cancer cells after 24 h of treatment with crude or infrared (IR)-treated jojoba oil at their respective IC_50_ concentrations. Bar graphs show relative mRNA expression of BCL2, BAX, and CASP3. These data report mRNA changes only and do not establish protein-level or functional activation of apoptosis. Data are presented as mean ± SD of biological replicates. Statistical significance versus untreated control: *** *p* < 0.001, ** *p* < 0.01, and * *p* < 0.05.

To determine whether the reduced cell viability and altered cell-cycle distribution were accompanied by changes in apoptosis-associated molecular markers, the relative mRNA expression of the anti-apoptotic gene *BCL2*, the pro-apoptotic gene *BAX*, and the executioner caspase gene *CASP3* was quantified by quantitative reverse-transcription polymerase chain reaction (qRT-PCR) (Table 10 and Figure 8). These measurements assess mRNA levels only and do not demonstrate protein abundance or functional activation of apoptotic pathways. Gene-expression values were normalized relative to the untreated control, for which the *BAX/BCL2* expression ratio was assigned a reference value of 1.0.

Treatment with crude jojoba oil significantly altered the expression of all three genes. Relative *BCL2* expression decreased to 0.77 ± 0.027 (*p* < 0.05), whereas *BAX* expression increased to 2.11 ± 0.194 (*p* < 0.05) and *CASP3* expression increased to 3.16 ± 0.221 (*p* < 0.05) compared with untreated cells. Consequently, the calculated *BAX/BCL2* mRNA expression ratio increased to 2.74. This transcriptional pattern, reduced BCL2 mRNA together with increased BAX and CASP3 mRNA, is consistent with a transcriptional response related to apoptosis, but it does not demonstrate apoptotic cell death or protein-level activation of apoptotic machinery.

A more pronounced transcriptional response was observed following treatment with IR-treated jojoba oil. Thermal control showed BAX 2.15 ± 0.20, BCL2 0.76 ± 0.03, CASP3 3.20 ± 0.23, and BAX/BCL2 ratio 2.80, similar to crude oil (*p* > 0.05), whereas IR-treated oil remained significantly different (BAX 3.67, BCL2 0.61, CASP3 5.73, ratio 6.02; *p* < 0.05) (Supplementary Table S1). Relative *BCL2* expression decreased to 0.61 ± 0.032, representing a significant reduction compared with both the untreated control (*p* < 0.01) and crude-oil treatment (*p* < 0.05). In contrast, *BAX* expression increased to 3.67 ± 0.231 (*p* < 0.01 versus control and crude oil), while *CASP3* expression increased to 5.73 ± 0.298 (*p* < 0.001 versus control; *p* < 0.05 versus crude oil). Accordingly, the *BAX/BCL2* mRNA expression ratio increased to 6.02, more than twice the ratio observed after crude-oil treatment. These findings indicate that IR-treated jojoba oil produced a stronger shift in the mRNA balance of apoptosis-related genes. This does not establish an apoptotic mechanism at the protein or functional level.

The gene-expression findings complement the cell-cycle and viability results. IR-treated jojoba oil produced the lowest IC_50_ against SK-OV-3 cells and the most pronounced accumulation of cells in G0/G1, together with marked depletion of the S-phase population. The concurrent downregulation of BCL2 mRNA and upregulation of BAX and CASP3 mRNA indicate that the enhanced antiproliferative response to IR-treated oil was accompanied by changes in the expression of apoptosis-related genes. However, these data do not establish activation of apoptotic pathways, because protein abundance, caspase-3 cleavage, and functional cell-death assays were not performed. Nevertheless, the relationship between cell-cycle disruption and apoptotic signaling is likely multifactorial, and the present data do not establish a direct causal sequence between G0/G1 accumulation and the observed transcriptional changes.

The increase in the *BAX/BCL2* expression ratio is particularly relevant because the balance between pro- and anti-apoptotic members of the *BCL-2* family plays an important role in regulating mitochondrial susceptibility to apoptotic stimuli. Likewise, increased CASP3 mRNA is compatible with, but not evidence of, engagement of downstream apoptotic machinery. Caspase-3 requires proteolytic cleavage for enzymatic activation, which was not measured here. However, the present measurements were performed at the mRNA level. Therefore, these results do not establish corresponding changes in *BAX* or *BCL-2* protein abundance, mitochondrial outer-membrane permeabilization, cytochrome *c* release, or cleavage and enzymatic activation of *caspase-3*. Confirmation of the intrinsic mitochondrial apoptotic pathway would require complementary protein- and functional-level analyses.

The stronger apoptosis-related transcriptional response observed with IR-treated jojoba oil also occurred alongside the pronounced compositional differences identified by FTIR and GC-MS. These included the detection of a peak tentatively assigned to 9-hexadecenoic acid, 9-octadecenyl ester, (Z,Z)-(27.45%), detection of 9-eicosyne (8.97%), and changes in the relative abundance of several unsaturated lipid-associated constituents. Bioactive lipids have previously been reported to influence pathways involved in cellular stress, proliferation, and programmed cell death [43]. It is therefore plausible that the altered chemical profile coincided with the stronger molecular response detected only in IR-treated jojoba oil. However, the current experiments do not establish a causal role for any individual GC-MS-detected constituent. The apoptosis-associated conclusions are based on mRNA expression and do not establish protein-level or functional activation of the intrinsic apoptotic pathway.

Taken together, the qRT-PCR findings demonstrate that IR treatment substantially enhanced the ability of jojoba oil to modulate apoptosis-associated gene expression in SK-OV-3 cells, characterized by suppression of *BCL2*, induction of *BAX* and *CASP3*, and a pronounced increase in the *BAX/BCL2* mRNA expression ratio. When integrated with the lower IC_50_ and marked G0/G1 accumulation observed in the preceding experiments, these findings indicate that IR treatment strengthens the in vitro antiproliferative response to jojoba oil and is accompanied by changes in the expression of apoptosis-related genes. These data do not establish an apoptotic mechanism. Further confirmation at the protein and functional levels, including BAX and BCL-2 protein quantification, caspase-3 cleavage and activity, mitochondrial membrane potential, cytochrome c release, and Annexin V/PI staining, will be necessary before activation of the mitochondrial apoptotic pathway can be claimed.

### 2.10. Study Limitations and Future Directions

Several limitations should be considered when interpreting the present findings. First, all biological experiments were conducted under in vitro conditions. Consequently, the observed antimicrobial, biofilm formation inhibition, protein denaturation, fibroblast scratch-closure, and anticancer effects do not establish therapeutic efficacy, safety, bioavailability, pharmacokinetic behavior, or clinical effectiveness. Future studies should therefore validate these findings in appropriate in vivo models of infected wounds, inflammation, and ovarian cancer, accompanied by comprehensive safety and toxicological assessment. Second, the GC-MS assignments were based on mass-spectral library matching and should therefore be regarded as tentative. GC-MS data were obtained from representative preparation rather than from independent replicates. Relative peak areas are therefore reported without standard deviations, and no statistical inference is drawn from GC-MS comparisons. The GC-MS results should be considered semi-quantitative and hypothesis-generating. Independent replicate analyses with authentic standards would be required to confirm the reported differences and to establish absolute concentrations.

The reported peak-area percentages represent relative chromatographic abundance rather than absolute concentrations, and individual compounds were not quantitatively confirmed using authentic standards. No internal standard or calibration curve was used; therefore, absolute quantification was not possible. Statements referring to increases, decreases, appearance, or un-detection of GC–MS peaks refer to relative chromatographic representation or detectability, not to absolute amounts. Consequently, the biological effects cannot be attributed to absolute changes in any individual constituent. Moreover, the present analytical data do not establish the specific chemical reactions responsible for the compositional differences observed after IR treatment. A thermal control matched to the measured IR temperature–time profile was included and did not reproduce the major chemical or biological changes observed after IR treatment, indicating that the effects were associated with IR treatment beyond bulk heating. However, localized microthermal effects or transient hot spots cannot be fully excluded, and the term ‘IR treatment’ is therefore used rather than claiming a purely non-thermal mechanism. Future studies should incorporate targeted compound confirmation, quantitative chemical analysis, complementary structural techniques, and careful temperature monitoring during irradiation. The well diffusion assay is a screening method and is strongly influenced by the diffusion characteristics of the test substance. Because jojoba oil is lipophilic and the antibiotic controls are hydrophilic, the inhibition zone diameters obtained for the oils cannot be directly compared with those of gentamicin or fluconazole. The positive controls confirm only that the assay conditions supported antimicrobial activity. Claims of equivalent or superior potency relative to standard antibiotics are therefore not made. MIC and MBC/MFC data provide the primary concentration-based comparison, and further time-kill or synergy assays would be required for direct potency comparisons. Third, only one IR-treatment condition was evaluated. Systematic investigation of irradiation intensity, exposure duration, wavelength range, and temperature will therefore be necessary to define optimal processing conditions. Such studies should also examine batch-to-batch reproducibility and chemical standardization to determine whether the observed biological enhancement can be consistently reproduced and translated to larger-scale processing. The antiproliferative activity against SK-OV-3 findings also requires further validation. The experiments were limited to a single ovarian cancer cell line, SK-OV-3, without parallel testing in an appropriate nonmalignant cell model; therefore, cancer-cell selectivity and a therapeutic window cannot be established from the present data. In addition, apoptosis-associated conclusions were based primarily on changes in mRNA expression. Future mechanistic studies should therefore include protein-level analysis of Bax, Bcl-2, and caspase-3, assessment of caspase activation, mitochondrial membrane potential, cytochrome *c* release, and additional markers of apoptotic cell death. Investigation of cell-cycle regulatory proteins, including cyclins, cyclin-dependent kinases, and their inhibitors, would also help clarify the molecular basis of the pronounced G0/G1 accumulation. The anti-inflammatory and fibroblast findings should likewise be interpreted within the limitations of the assays used. The bovine serum albumin denaturation assay is a preliminary screening model and does not establish modulation of specific inflammatory pathways. Future studies should therefore investigate relevant inflammatory mediators and signaling pathways, including NF-κB and cyclooxygenase/lipoxygenase-related mechanisms. Similarly, the scratch assay represents a simplified model of fibroblast-mediated gap closure and does not reproduce the complexity of wound healing in vivo. Additional studies should distinguish the respective contributions of migration and proliferation and assess extracellular matrix deposition, collagen production, angiogenesis, inflammatory responses, and tissue remodeling in more physiologically representative models. Finally, formulation development may be necessary to improve stability, dispersibility, skin retention, and controlled delivery of IR-treated jojoba oil. Topical creams, hydrogels, nanoemulsions, and related delivery systems merit investigation. Evaluation of combinations with conventional antimicrobial or antiproliferative activity against SK-OV-3 agents may also determine whether additive or synergistic interactions occur and whether effective concentrations can be reduced. Together, these studies will be essential for defining the mechanisms, reproducibility, safety, and translational potential of IR-treated jojoba oil.

## 3. Materials and Methods

### 3.1. Jojoba Oil and Infrared Treatment

Cold-pressed, pure jojoba oil (*Simmondsia chinensis*) was obtained from Desert Whale Jojoba Company (Tucson, AZ, USA; Lot No. JW-2208). Before experimentation, the oil was stored in amber glass bottles under a nitrogen atmosphere at 4 °C to minimize exposure to light and oxidative deterioration. For infrared (IR) treatment, 15 mL of jojoba oil was transferred into an uncovered sterile 90 mm borosilicate glass Petri dish (Corning Inc., Corning, NY, USA). The sample was exposed to an IR lamp (Philips IR-R, 250 W; Eindhoven, The Netherlands) positioned 20 cm above the oil surface. The incident power density at the sample surface was 50 mW/cm^2^. The oil temperature was monitored continuously with a calibrated K-type thermocouple (accuracy ±0.5 °C) placed at the center of the oil volume. Under these conditions, the oil temperature increased from 25.0 ± 0.5 °C to 58.3 ± 1.2 °C over 30 min. A thermal control was prepared by heating 15 mL of jojoba oil in a thermostatic water bath using the same temperature–time profile (25.0–58.1 ± 1.0 °C over 30 min) without IR exposure. Preliminary optimization identified 30 min as the shortest exposure producing maximal IR-specific FTIR changes and antimicrobial activity (Supplementary Table S3). Therefore, 30 min was selected for all subsequent experiments. Treatment duration was selected on the basis of comparative changes in the FTIR carbonyl profile and antimicrobial activity. A 30 min exposure produced the most suitable overall response under the tested conditions and was therefore selected for subsequent experiments. Oil subjected to this treatment is hereafter referred to as IR-treated jojoba oil, whereas untreated oil processed without IR exposure served as the crude jojoba oil control. Immediately after treatment, both crude and IR-treated oil samples were transferred to appropriate containers and stored at −20 °C under an argon atmosphere until subsequent chemical and biological analyses to minimize post-treatment oxidative changes [44]. The oil temperature was monitored continuously using a calibrated K-type thermocouple (accuracy ±0.5 °C) placed at the center of the oil volume. Under the defined IR conditions (30 min, 50 mW/cm^2^, 20 cm distance), the oil temperature increased from 25.0 ± 0.5 °C to 58.3 ± 1.2 °C over 30 min. A thermal control was prepared by heating 15 mL of jojoba oil in a thermostatic water bath using the same temperature–time profile (25.0–58.1 ± 1.0 °C over 30 min) without IR exposure. The thermal control oil was then subjected to the same FTIR, GC-MS, and biological assays as crude and IR-treated oils. Compatible thermal control results have been reported in Supplementary Table S1.

### 3.2. Fourier Transform Infrared Spectroscopy

Fourier transform infrared (FTIR) spectra of crude and infrared (IR)-treated jojoba oils were recorded using a Bruker Alpha II spectrometer (Bruker Optics, Billerica, MA, USA) equipped with a single-bounce diamond attenuated total reflectance (ATR) accessory. For each measurement, approximately 15 µL of oil was placed directly onto the ATR crystal, and spectra were acquired over the range of 4000–400 cm^−1^ at a spectrum of 4 cm^−1^ using 64 accumulated scans. An air-background spectrum was collected before each sample measurement. Spectral processing was performed using OPUS software version 8.5 (Bruker), including baseline correction and normalization. Three independently prepared samples were analyzed for each oil type under identical instrumental conditions. Representative spectra are presented for comparison [45].

### 3.3. Gas Chromatography-Mass Spectrometry (GC-MS) Analysis

The chemical profiles of crude and infrared (IR)-treated jojoba oils were characterized by GC-MS using a Shimadzu GC-2030 gas chromatograph coupled to a TQ8040 NX triple-quadrupole mass spectrometer (Shimadzu Corp., Kyoto, Japan). For analysis, 25 mg of each oil sample was dissolved in 1 mL of HPLC-grade *n*-hexane (Merck, Darmstadt, Germany) and filtered through a 0.22 µm polytetrafluoroethylene (PTFE) syringe filter (Pall Corp., Port Washington, NY, USA) before injection. Chromatographic separation was performed using an Rxi-5Sil MS capillary column (30 m × 0.25 mm internal diameter, 0.25 µm film thickness; Restek, Bellefonte, PA, USA). The oven temperature was initially maintained at 50 °C for 2 min, increased to 300 °C at a rate of 8 °C/min, and held at 300 °C for 10 min. The injector temperature was maintained at 260 °C, and samples were injected at a volume of 1 µL using a split ratio of 1:30. High-purity helium (99.999%) was used as the carrier gas at a constant flow rate of 1.5 mL/min. Mass-spectral acquisition was performed using electron ionization at 70 eV over an *m*/*z* range of 45–650. The ion-source and interface temperatures were maintained at 230 and 280 °C, respectively. Compounds with match scores < 80 or RI deviations > 15 units were considered low-confidence and were either removed or explicitly labeled as tentative. All GC-MS assignments should be regarded as tentative unless confirmed with authentic standards (Supplementary Table S5). GC-MS data are reported as relative peak areas (%). These values represent relative abundance within each sample and are not absolute concentrations. Therefore, changes in relative peak areas are described as relative enrichment or depletion and should not be interpreted as absolute increases, decreases, generation, or degradation of compounds. Quantitative confirmation would require calibration with authentic standards, which was not performed in this study. All GC-MS assignments should be regarded as tentative unless confirmed with authentic standards. Retention indices were determined using a C7–C40 *n*-alkane standard mixture (Merck) analyzed under the same chromatographic conditions [46]. Compound assignments were considered tentative because authentic reference standards were not used for structural confirmation. Peak-area percentages are normalized to the total detected chromatographic area and do not represent absolute concentration. Therefore, differences in relative peak areas cannot be interpreted as evidence of absolute increases or decreases in individual constituents.

### 3.4. Agar Well Diffusion Assay

The antimicrobial activity of crude and infrared (IR)-treated jojoba oils was initially evaluated using the agar well diffusion method [47]. The tested microorganisms included *Enterococcus faecalis* (ATCC 29212), *Staphylococcus aureus* (ATCC 29213), *Salmonella Typhi*, *Pseudomonas aeruginosa* (ATCC 27853), and *Candida albicans* (ATCC 10231). Each microorganism was cultured overnight in an appropriate growth broth, and the resulting suspension was adjusted to a turbidity equivalent to 0.5 McFarland using a DensiCHEK™ Plus turbidimeter (bioMérieux, Marcy-l’Étoile, France). Standardized microbial suspensions were uniformly inoculated onto Mueller–Hinton agar (MHA) plates for bacterial strains and Sabouraud dextrose agar (SDA) plates for *C. albicans* using sterile cotton swabs. Wells measuring 8 mm in diameter were aseptically prepared using a sterile cork borer (Fisher Scientific, Hampton, NH, USA). Each well was loaded with 50 µL of crude jojoba oil or IR-treated jojoba oil, each prepared at 500 mg/mL in 1% dimethyl sulfoxide (DMSO), corresponding to 25 mg oil per well. Gentamicin (10 µg/well) was used as the positive control for bacterial strains, fluconazole (25 µg/well) as the positive control for *C. albicans*, and 1% DMSO as the negative control. Because jojoba oil is lipophilic and the antibiotic controls are hydrophilic, inhibition zone diameters are not directly comparable and are reported only as an initial screening of antimicrobial activity under the defined assay conditions. Following sample application, the plates were maintained at 4 °C for 2 h to facilitate radial diffusion of the test preparations into the agar before incubation. Bacterial plates were incubated at 37 °C for 24 h, whereas *C. albicans* plates were incubated at 30 °C for 48 h. The antimicrobial activity was expressed as the diameter of the inhibition zone, measured in millimeters using a digital Vernier caliper (Mitutoyo, Kawasaki, Japan). Measurements included the 8 mm well diameter.

### 3.5. Minimum Inhibitory Concentration (MIC) and Minimum Bactericidal/Fungicidal Concentration (MBC/MFC)

The minimum inhibitory concentrations (MICs) of crude and infrared (IR)-treated jojoba oils were determined using a broth microdilution method with reference to Clinical and Laboratory Standards Institute (CLSI) guidelines for bacteria and yeast. Stock preparations of crude and IR-treated jojoba oils were prepared at 500 mg/mL using dimethyl sulfoxide (DMSO; Sigma-Aldrich, St. Louis, MO, USA). Twofold serial dilutions were prepared in sterile 96-well flat-bottom microplates (Costar^®^ 3799; Corning Inc., Corning, NY, USA) using Mueller–Hinton broth (MHB) for bacterial strains and Sabouraud dextrose broth (SDB) for *C. albicans*. For each assay, 100 µL of the appropriate oil dilution was dispensed into the designated wells, followed by 100 µL of the standardized microbial suspension. The inoculum was adjusted to approximately 5 × 10^5^ CFU/mL and verified spectrophotometrically at 625 nm using a BioPhotometer™ D30 (Eppendorf, Hamburg, Germany). Appropriate growth and sterility controls were included in each experiment. The vehicle control contained the corresponding concentration of DMSO without jojoba oil, whereas uninoculated sterile medium served as the sterility control. Plates were incubated under the conditions specified for each microorganism. Following incubation, 20 µL of 0.01% (*w*/*v*) resazurin solution (Sigma-Aldrich) was added to each well, and the plates were incubated for an additional 2 h. The MIC was defined as the lowest concentration of jojoba oil that prevented the resazurin color change from blue to pink, indicating the absence of detectable microbial metabolic activity. To determine the minimum bactericidal concentration (MBC) or minimum fungicidal concentration (MFC), 10 µL aliquots were collected from wells showing no visible growth and inoculated onto Mueller–Hinton agar for bacteria or Sabouraud dextrose agar for *C. albicans*. Following incubation, the MBC or MFC was defined as the lowest oil concentration producing ≥99.9% reduction in viable microorganisms relative to the initial inoculum [48]. MIC values were determined from three independent experiments, each performed in triplicate. Technical replicates were averaged within each independent experiment; only independent experiments were used for statistical analysis. MIC values are reported as median (range) because MICs are discrete twofold dilutions. The primary inferential test for the global comparison between crude and IR-treated oils across the five microorganisms was the exact two-sided Wilcoxon signed-rank test, because no directional hypothesis was pre-specified. One-sided values are reported only as directional sensitivity analyses and are not used as significance claims. With five paired microorganisms, the smallest attainable two-sided exact *p*-value is 0.0625; the global comparison is therefore inherently limited in power and is interpreted accordingly. Per-microorganism comparisons were performed using paired *t*-tests on log_2_-transformed MIC values from independent experiments and were adjusted for multiple testing using the Benjamini–Hochberg procedure; these are secondary/exploratory analyses that do not override the primary global test and are not presented as establishing global significance. MBC/MIC and MFC/MIC ratios were calculated from median values. The global and per-microorganism analyses use different tests by design: the exact two-sided Wilcoxon signed-rank test is the primary inferential test for the global comparison, and paired *t*-tests on log_2_-transformed values are used only for the secondary, exploratory per-microorganism comparisons.

### 3.6. Biofilm Inhibition Assay

The effects of crude and infrared (IR)-treated jojoba oils on biofilm formation were evaluated using the crystal violet staining assay [49]. Standardized bacterial suspensions were prepared at approximately 1 × 10^6^ CFU/mL in Mueller–Hinton broth (MHB) supplemented with 1% glucose and dispensed into sterile 96-well microplates. Each bacterial strain was exposed to sub-bactericidal concentrations of crude or IR-treated jojoba oil corresponding to 25%, 50%, and 75% of the respective minimum bactericidal concentration (MBC). Untreated bacterial cultures serve as biofilm-growth controls. Following incubation at 37 °C for 24 h, the culture medium containing planktonic cells was carefully removed, and the wells were washed three times with phosphate-buffered saline (PBS; pH 7.4; Gibco, Thermo Fisher Scientific, Waltham, MA, USA) to remove nonadherent cells. The remaining adherent biofilms were fixed with methanol for 20 min and subsequently stained with 0.1% (*w*/*v*) crystal violet for 15 min. Excess stain was removed by repeated washing with water, after which the biofilm-bound crystal violet was solubilized using 33% glacial acetic acid. Absorbance was measured at 595 nm using a Synergy™ H1 microplate reader (BioTek Instruments, Winooski, VT, USA). Biofilm inhibition was calculated relative to the untreated biofilm-growth control and expressed as percentage inhibition. This assay measures adherent biofilm biomass (cells and extracellular matrix) but cannot distinguish between inhibiting new formation and eradicating mature biofilms. Bacterial suspensions of 1 × 10^6^ CFU/mL in MHB with 1% glucose were added to 96-well microplates and exposed to crude or IR-treated jojoba oil at sub-MBC levels (25%, 50%, and 75% MBC) throughout a 24 h formation period. Thus, the assay evaluates biofilm inhibition rather than eradication. Controls included medium-only blanks (negative), untreated cultures (growth control), and sub-MBC gentamicin (positive).

Following a 24 h incubation at 37 °C, planktonic cells were removed, and wells were washed three times with PBS. Adherent biofilms were fixed with methanol for 20 min and stained with 0.1% crystal violet for 15 min. After washing away excess stains, biofilm-bound crystal violet was solubilized with 33% glacial acetic acid, and absorbance was measured at 595 nm. The assay comprised three independent experiments, each using six technical replicates that were averaged per experiment.

### 3.7. Protein Denaturation Inhibition Assay

The ability of crude and IR-treated jojoba oils to inhibit protein denaturation was evaluated using the heat-induced bovine serum albumin (BSA) denaturation assay [36]. This is a cell-free preliminary screening assay that measures the ability of a sample to inhibit heat-induced protein denaturation. It does not establish anti-inflammatory activity at the cellular or organismal level and does not identify specific inflammatory mediators or signaling pathways. BSA (Sigma-Aldrich, St. Louis, MO, USA) was prepared at 1% (*w*/*v*) in phosphate-buffered saline (PBS; pH 6.4). Crude and IR-treated jojoba oils were prepared at the required test concentrations using 0.5% dimethyl sulfoxide (DMSO) as the vehicle. Diclofenac sodium was included as the positive control. For each assay, the appropriate volume of the test preparation was combined with BSA solution and PBS. The reaction mixtures were initially incubated at 37 °C for 20 min and subsequently heated at 57 °C for 3 min to induce protein denaturation. Following cooling to room temperature, PBS was added as specified in the assay protocol, and the resulting turbidity was measured at 660 nm using a UV-1900i UV-visible spectrophotometer (Shimadzu, Kyoto, Japan). Protein denaturation inhibition was calculated relative to the untreated denaturation control according to the following equation:

$$\mathrm{Inhibition}\;(\%)\;=[\frac{\left(A_{\mathrm{control}}\;-\;A_{\mathrm{sample}}\right)}{A_{\mathrm{control}}}]\;\times \;100$$

where *A_control_* represents the absorbance of the denaturation control and *A_sample_* represents the absorbance measured in the presence of crude or IR-treated jojoba oil. The concentration required to inhibit protein denaturation by 50% (IC_50_) was subsequently determined from the concentration–response data.

### 3.8. Fibroblast Scratch Closure Assay

The effect of crude and infrared (IR)-treated jojoba oils on fibroblast-mediated scratch closure was evaluated using an in vitro scratch assay [50]. Human dermal fibroblasts (HDFa; ATCC^®^ PCS-201-012™) were obtained from Science Way for Scientific Researches and Consultations (Cairo, Egypt) and maintained in high-glucose Dulbecco’s modified Eagle medium (DMEM; Gibco) supplemented with 10% fetal bovine serum (FBS; HyClone™, GE Healthcare, Chicago, IL, USA) and 1% penicillin–streptomycin (Gibco). Cells were cultured at 37 °C in a humidified atmosphere containing 5% CO_2_ using a Heracell™ 150i incubator. HDFa cells were seeded into 12-well culture plates at a density of 2 × 10^5^ cells/well and allowed to form a confluent monolayer. A uniform linear scratch was then generated across each monolayer using a sterile 200 µL pipette tip. Detached cells and cellular debris were carefully removed by washing with phosphate-buffered saline (PBS). The scratched monolayers were subsequently incubated in serum-free DMEM containing crude or IR-treated jojoba oil at the selected noncytotoxic concentration. Vehicle-treated cells containing the corresponding concentration of dimethyl sulfoxide (DMSO) served as the untreated control. Representative images of the same scratch regions were acquired immediately after scratch formation (0 h) and after 24 h of treatment using an inverted Nikon Eclipse Ti2 microscope equipped with a digital camera. The cell-free areas were quantified using ImageJ software version 1.54 (National Institutes of Health, Bethesda, MD, USA). Scratch closure was calculated according to the following equation:

$$\mathrm{Scratch}\;\mathrm{closure}\;(\%)\;=\;[\frac{\left(A_{0}\;-\;A_{t}\right)}{A_{0}}]\;\times \;100$$

where *A*_0_ represents the initial cell-free area at 0 h and *A_t_* represents the residual cell-free area after 24 h of treatment. The migration rate was subsequently calculated from the change in scratch width over the 24 h observation period.

### 3.9. Antiproliferative Activity Against SK-OV-3 Ovarian Cancer Cells

The effects of crude and infrared (IR)-treated jojoba oils on ovarian cancer cell viability were evaluated using the MTT assay [51]. SK-OV-3 human ovarian cancer cells (ATCC^®^ HTB-161™) were obtained from Science Way for Scientific Researches and Consultations (Cairo, Egypt) and maintained in RPMI-1640 medium (Gibco) supplemented with 20% fetal bovine serum (FBS; HyClone™), 1% penicillin–streptomycin, and 0.01 mg/mL bovine insulin (Sigma-Aldrich). Cells were cultured at 37 °C in a humidified atmosphere containing 5% CO_2_. For the cell-viability assay, SK-OV-3 cells were seeded into 96-well culture plates at a density of 1.5 × 10^4^ cells/well and allowed to adhere overnight. Cells were subsequently exposed to increasing concentrations of crude or IR-treated jojoba oil (15.6–1000 µg/mL) prepared in RPMI-1640 medium containing 0.1% dimethyl sulfoxide (DMSO). Vehicle-treated cells containing the corresponding concentration of DMSO without jojoba oil served as the untreated control. Following 24 h of exposure, 20 µL of MTT solution (5 mg/mL in phosphate-buffered saline; Sigma-Aldrich, M5655) was added to each well, and the plates were incubated for an additional 4 h. After incubation, the resulting formazan crystals were solubilized in 150 µL of DMSO, and absorbance was measured at 570 nm with a reference wavelength of 630 nm using a Synergy™ H1 microplate reader (BioTek Instruments). Cell viability was expressed relative to the vehicle-treated control. Concentration-response curves were generated, and the concentration required to reduce cell viability by 50% (IC_50_) was estimated by nonlinear regression using GraphPad Prism version 9.5.0 (GraphPad Software, San Diego, CA, USA).

### 3.10. Cell-Cycle Distribution Analysis

Cell-cycle distribution was analyzed by propidium iodide (PI) staining and flow cytometry [17]. SK-OV-3 cells were seeded into 6-well culture plates at a density of 5 × 10^5^ cells/well and allowed to adhere overnight. Cells were subsequently exposed for 24 h to crude or infrared (IR)-treated jojoba oil at their respective IC_50_ concentrations, as determined by the 24 h MTT assay. Vehicle-treated cells containing 0.1% dimethyl sulfoxide (DMSO) served as the control. Following treatment, the culture medium was removed, and adherent cells were detached using 0.25% trypsin-EDTA (Gibco). Cell suspensions were collected by centrifugation at 300× *g* for 5 min, and the resulting pellets were washed twice with ice-cold phosphate-buffered saline (PBS). Cells were fixed by the gradual addition of pre-chilled 70% ethanol (−20 °C) while gently vortexing to minimize cell aggregation and were subsequently maintained at −20 °C overnight. The following day, fixed cells were collected by centrifugation and washed with PBS to remove residual ethanol. Cells were then stained with a freshly prepared solution containing 50 µg/mL PI, 100 µg/mL RNase A, and 0.1% Triton X-100 (Sigma-Aldrich). Staining was performed for 30 min at 37 °C in the dark. Cellular DNA content was measured using a BD FACSCanto™ II flow cytometer (BD Biosciences, San Jose, CA, USA) equipped with a 488 nm laser. A total of 20,000 events were acquired for each sample. Flow-cytometric data were analyzed using FlowJo™ software version 10.8 (BD Biosciences), and DNA-content histograms were used to determine the relative percentages of cells in the G0/G1, S, and G2/M phases of the cell cycle.

### 3.11. Quantification of Apoptosis-Related Gene Expression

Changes in the expression of apoptosis-associated genes were evaluated in SK-OV-3 cells following 24 h of exposure to crude or infrared (IR)-treated jojoba oil at their respective IC_50_ concentrations. Vehicle-treated cells served as the control. Total RNA was isolated using the RNeasy Plus Mini Kit (Qiagen, Hilden, Germany; Cat. No. 74134) according to the manufacturer’s instructions. Genomic DNA was removed using the gDNA Eliminator column supplied with the kit. RNA concentration and purity were assessed using a NanoDrop™ One spectrophotometer (Thermo Fisher Scientific) by measuring absorbance at 260 nm and determining the A_260_/A_280_ ratio, with values ≥1.9 considered indicative of acceptable RNA purity. For complementary DNA (cDNA) synthesis, 1 µg of total RNA from each sample was reverse-transcribed using the High-Capacity cDNA Reverse Transcription Kit (Applied Biosystems, Foster City, CA, USA; Cat. No. 4368814) with random primers. Reverse transcription was performed in a T100™ thermal cycler (Bio-Rad Laboratories) according to the manufacturer’s recommended protocol. Quantitative real-time PCR was performed using a CFX96 Touch™ Real-Time PCR Detection System (Bio-Rad Laboratories) and PowerUp™ SYBR™ Green Master Mix (Applied Biosystems; Cat. No. A25742). Each 10 µL reaction contained 5 µL of 2× SYBR Green Master Mix, forward and reverse primers at a final concentration of 0.5 µM each, 2 µL of diluted cDNA corresponding to approximately 50 ng of input RNA, and nuclease-free water to the final reaction volume. Primer sequences for *BAX*, *BCL2*, *CASP3*, and the reference gene *GAPDH* are provided in Table 11. Amplification conditions consisted of an initial incubation at 50 °C for 2 min and activation at 95 °C for 2 min, followed by 40 cycles of denaturation at 95 °C for 15 s and annealing/extension at 60 °C for 60 s. Following amplification, melting-curve analysis was performed from 65 to 95 °C using 0.5 °C increments to assess amplification specificity and verify the presence of a single predominant amplification product. qRT-PCR was performed on three independent biological experiments (*n* = 3 independent cell cultures). Each biological sample was analyzed in technical triplicate. The median of technical triplicates was used as the value for each biological replicate. Statistical analysis was performed on the biological replicate values, not on technical replicates. Gene expression was normalized to GAPDH, and relative expression was calculated using the 2^−ΔΔCT^ method. Expression levels were reported as fold changes relative to the vehicle-treated control, which was assigned a value of 1.00 [52]. These measurements quantify mRNA levels only. They do not establish corresponding changes in protein abundance, caspase-3 cleavage or enzymatic activity, mitochondrial outer-membrane permeabilization, cytochrome c release, or functional apoptotic cell death.

### 3.12. Data Handling and Statistical Analysis

Unless otherwise noted, experiments were performed in three independent biological replicates (*n* = 3). Technical replicates were averaged within each biological replicate and were not treated as independent observations. Because the Shapiro–Wilk test has limited power with *n* = 3, normality could not be reliably established, and statistical results should be interpreted accordingly. Tests were therefore chosen according to data type and the number of comparison groups, as specified below.

Antimicrobial endpoints (MIC and MBC/MFC). Because these data are discrete, ordinal, and paired across five microorganisms, the primary global comparison used the exact two-sided Wilcoxon signed-rank test; no directional hypothesis was pre-specified. One-sided values (*p* = 0.0313) are reported only as directional sensitivity analyses, not as significance claims. Both global comparisons returned *p* = 0.0625 and are described as trends; with five paired microorganisms, 0.0625 is the minimum attainable two-sided exact *p*-value. Per-microorganism MIC comparisons were secondary/exploratory and were performed using paired *t*-tests on log_2_-transformed MIC values with Benjamini–Hochberg adjustment, as described in Section 3.5 and reported in Table 4. Per-microorganism MBC/MFC comparisons are reported descriptively as median (range) with exploratory BH-adjusted *p*-values. None of the per-microorganism analyses override the primary global test. No ANOVA- or Tukey-based significance claim is made for MIC, MBC, or MFC data.

Cell-based assays with one factor and three groups (scratch closure, cell-cycle distribution, and qRT-PCR). Comparisons among the untreated control, crude jojoba oil, and IR-treated jojoba oil were performed using one-way ANOVA followed by Tukey’s HSD post hoc test, with adjusted *p* < 0.05 considered statistically significant, as stated in the corresponding table and figure legends. Given *n* = 3, these comparisons should be regarded as exploratory.

Concentration-response assays (MTT viability and BSA denaturation). Concentration-specific comparisons were performed using two-way ANOVA followed by Sidak’s multiple-comparison test. IC_50_ values were estimated by nonlinear regression and compared using the extra sum-of-squares F-test, as stated in the corresponding table and figure legends.

## 4. Conclusions

This study indicates that infrared (IR) treatment can substantially modify the physicochemical properties and GC-MS-detectable chemical profile of jojoba oil and is associated with enhanced in vitro biological effects. Fourier-transform infrared (FTIR) spectroscopy revealed spectral changes in regions associated with ester carbonyl groups, unsaturation, hydroxyl interactions, and long-chain hydrocarbon structures, whereas gas chromatography-mass spectrometry (GC-MS) revealed qualitative and relative compositional differences between crude and IR-treated jojoba oils. These chemical changes coincided with enhanced antimicrobial and antibiofilm activities, greater inhibition of protein denaturation in a cell-free assay, and improved fibroblast scratch closure in vitro. Furthermore, IR treatment enhanced the in vitro antiproliferative and cytotoxic activity of jojoba oil against SK-OV-3 ovarian cancer cells, as demonstrated by a lower IC_50_ value, pronounced accumulation of cells in the G0/G1 phase, and changes in the expression of apoptosis-related genes, including increased BAX and CASP3 mRNA expression, decreased BCL2 mRNA expression, and an increased BAX/BCL2 mRNA expression ratio. However, the present study does not establish which specific chemical changes are responsible for the observed biological effects, and no in vivo experimental data are available. Moreover, the findings obtained from SK-OV-3 cells alone do not establish jojoba oil as an anticancer therapeutic agent or a therapeutic candidate. Collectively, these findings are limited to specific in vitro biological effects and should be regarded as a starting point for further mechanism, safety, and in vivo investigations. The principal novelty of this study lies in the integrated evaluation of IR treatment-associated chemical modifications alongside antimicrobial, antibiofilm, protein denaturation, fibroblast scratch-closure, and SK-OV-3 cell responses within a single experimental framework, rather than in establishing therapeutic efficacy or a specific molecular mechanism. Further validation, particularly through mechanistic investigations, safety assessments, and in vivo studies, will be necessary before any potential biomedical, pharmaceutical, or dermatological applications can be considered.

## Figures and Tables

**Table 1:** * GC-MS-detectable chemical profile of crude (non-irradiated) jojoba oil. Tentatively identified compounds are presented with their retention time (RT, min), molecular weight (MW), molecular formula, relative chromatographic peak area (%), and chemical class. Compound assignments were based on mass-spectral library matching, and the reported peak-area percentages represent relative chromatographic abundance rather than absolute concentrations.

Compound Name	RT (min)	MW	Molecular Formula	Relative Peak Area (%)	Class
Oxiraneundecanoic acid, 3-pentyl-, methyl ester, cis	28.82	312	C_19_H_36_O_3_	1.01	Epoxy fatty acid ester
cis-13-Eicosenoic acid	29.46	310	C_20_H_38_O_2_	6.34	Fatty acid
9-Octadecenoic acid (Z)-	29.60	282	C_18_H_34_O_2_	3.43	Fatty acid
17-Octadecynoic acid	31.07	280	C_18_H_32_O_2_	6.96	Fatty acid (alkyne)
1-Heptatriacotanol	33.52	536	C_37_H_76_O	5.80	Fatty alcohol
13-Heptadecyn-1-ol	34.39	252	C_17_H_32_O	10.66	Fatty alcohol (alkyne)
Friedelan-3-one	35.53	426	C_30_H_50_O	2.87	Triterpenoid (ketone)
Isochiapin B	41.66	346	C_19_H_22_O_6_	0.58	Sesquiterpene lactone
β-Sitosterol	44.30	414	C_29_H_50_O	19.95	Phytosterol (steroid)

* Peak areas are normalized to the total detected chromatographic area and are not absolute concentrations. Relative peak-area changes do not establish absolute increases or decreases in individual compounds. Only compounds meeting the stated identification criteria are listed. Relative peak areas are not renormalized after exclusion.

**Table 2:** * GC-MS-detectable chemical profile of infrared (IR)-treated jojoba oil following 30 min of exposure at 50 mW/cm^2^. Tentatively identified compounds are presented with their retention time (RT, min), molecular weight (MW), molecular formula, relative chromatographic peak area (%), and chemical class. Reported peak-area percentages represent relative chromatographic abundance rather than absolute concentration.

Compound Name	RT (min)	MW	Molecular Formula	Relative Peak Area (%)	Class
9-Eicosyne	31.07	278	C_20_H_38_	8.97	Hydrocarbon (alkyne)
9-Octadecen-1-ol, (Z)-	34.37	268	C_18_H_36_O	9.33	Fatty alcohol
13-Heptadecyn-1-ol	36.19	252	C_17_H_32_O	2.02	Fatty alcohol (alkyne)
9-Octadecenoic acid (Z)-	40.72	282	C_18_H_34_O_2_	2.28	Fatty acid
1-Heptatriacotanol	43.83	536	C_37_H_76_O	3.96	Fatty alcohol
β-Sitosterol	44.30	414	C_29_H_50_O	21.47	Phytosterol (steroid)
9-Hexadecenoic acid, 9-octadecenylester, (Z,Z)-	44.85	504	C_34_H_64_O_2_	27.45	Fatty acid ester
cis-13-Eicosenoic acid	44.95	310	C_20_H_38_O_2_	18.17	Fatty acid
cis-10-Nonadecenoic acid	45.26	296	C_19_H_36_O_2_	4.09	Fatty acid

* Peak areas are normalized to the total detected chromatographic area and are not absolute concentrations. Relative peak-area changes do not establish absolute increases or decreases in individual compounds. Only compounds meeting the stated identification criteria are listed. Relative peak areas are not renormalized after exclusion.

**Table 3:** Inhibition zone diameters of untreated and infrared (IR)-treated jojoba oils against the tested microorganisms. Values are presented as mean ± standard deviation (SD) from three independent experiments, each performed in triplicate, with technical replicates averaged within each independent experiment. The reported inhibition zone diameters include the diameter of the well. Both crude and IR-treated jojoba oils were applied at 25 mg/well. Gentamicin (10 µg/well) was used as the positive control for bacterial strains, whereas fluconazole (25 µg/well) served as the positive control for *C. albicans*. Because jojoba oil is lipophilic, whereas the antibiotic controls are hydrophilic, the inhibition zone diameters are not directly comparable. Statistical comparisons between crude and IR-treated jojoba oils were performed separately for each microorganism using paired *t*-tests. Both raw and Benjamini–Hochberg (BH)-adjusted *p*-values are presented, with adjusted *p* < 0.05 considered statistically significant. The positive control was gentamicin for bacterial strains and fluconazole for yeast. All reported *p*-values refer exclusively to comparisons between the two oil treatments.

Microorganism	Untreated Oil (mm)	IR-Treated Oil (mm)	Raw *p*	BH-Adjusted *p*
*Enterococcus faecalis*	15 ± 0.5	17 ± 0.2	0.0097	0.0121 *
*Staphylococcus aureus*	21 ± 0.3	25 ± 0.7	0.0015	0.0060 **
*Salmonella typhi*	21 ± 0.7	23 ± 0.5	0.0213	0.0213 *
*Pseudomonas aeruginosa*	12 ± 0.4	17 ± 0.9	0.0038	0.0063 **
*Candida albicans*	24 ± 0.3	30 ± 0.2	0.0012	0.0060 **

Data are presented as the mean ± SD of biological replicates. Statistical significance is indicated by ** for *p* < 0.01 and * for *p* < 0.05.

**Table 4:** Minimum inhibitory concentrations (MICs) of crude, thermally controlled, and infrared (IR)-treated jojoba oils against the tested microorganisms. Values are presented as the median (range) from three independent experiments (*n* = 3). MIC values correspond to discrete twofold serial dilution steps. Fold reduction was calculated as MIC of crude oil divided by MIC of IR-treated oil (MIC_crude/MIC_IR-treated). The primary global comparison was the exact two-sided Wilcoxon signed-rank test (*p* = 0.0625; trend). Per-microorganism paired *t*-tests on log_2_-transformed MIC values are secondary/exploratory and BH-adjusted; they do not establish global significance.

Microorganism	Untreated MIC Median (Range) μg/mL	Thermal Control MIC (µg/mL)	IR-Treated MIC Median (Range) μg/mL	Fold Reduction
*Enterococcus faecalis*	62.5 (62.5–62.5)	62.5	31.25 (31.25–31.25)	2
*Staphylococcus aureus*	31.25 (31.25–31.25)	31.25	15.62 (15.62–15.62)	2
*Salmonella typhi*	31.25 (31.25–31.25)	31.25	15.62 (15.62–15.62)	2
*Pseudomonas aeruginosa*	62.5 (62.5–62.5)	62.5	15.62 (15.62–15.62)	4
*Candida albicans*	15.62 (15.62–15.62)	15.62	7.8 (7.8–7.8)	2

Fold reduction = MIC_crude/MIC_IR-treated. The primary global comparison was the exact two-sided Wilcoxon signed-rank test (*p* = 0.0625; trend). Per-microorganism comparisons are secondary/exploratory and were performed using paired *t*-tests on log2-transformed MIC values from the three independent experiments, with Benjamini–Hochberg adjustment. Raw and BH-adjusted per-microorganism *p*-values are given in the footnote table below. These exploratory analyses do not override the primary global test and are not presented as establishing global significance.

**Table 5:** Minimum bactericidal concentrations (MBCs) and minimum fungicidal concentrations (MFCs) of crude and infrared (IR)-treated jojoba oils against the tested microorganisms. Values are presented as the median (range) from three independent experiments, each performed in triplicate. Technical replicates were not treated as independent observations in the statistical analyses. MBC/MIC and MFC/MIC ratios were calculated using the corresponding median values. The overall treatment effect was assessed using the exact two-sided Wilcoxon signed-rank test, the pre-specified primary test for these discrete, ordinal data. All five paired differences were in the direction of lower MBC/MFC values after IR treatment; because 0.0625 is the minimum attainable two-sided exact *p*-value with five paired microorganisms, the overall effect is reported as a consistent trend (*p* = 0.0625) and is not described as statistically significant. Per-microorganism comparisons are reported descriptively as median (range) and as BH-adjusted exploratory *p*-values; they are not presented as establishing global significance, and no ANOVA- or Tukey-based significance claim is made.

Microorganism	Untreated MBC/MFC Median (Range) μg/mL	Thermal Control MBC/MFC (µg/mL)	IR-Treated MBC/MFC Median (Range) μg/mL	Untreated MBC/MIC	IR-Treated MBC/MIC
*Enterococcus faecalis*	125 (125–125)	125	62.5 (62.5–62.5)	2	2
*Staphylococcus aureus*	62.5 (62.5–62.5)	62.5	31.25 (31.25–31.25)	2	2
*Salmonella typhi*	62.5 (62.5–62.5)	62.5	31.25 (31.25–31.25)	2	2
*Pseudomonas aeruginosa*	62.5 (62.5–62.5)	62.5	31.25 (31.25–31.25)	1	2
*Candida albicans*	31.25 (31.25–31.25)	31.25	15.62 (15.62–15.62)	2	2

Overall paired exact Wilcoxon signed-rank test (primary, two-sided): *p* = 0.0625 (minimum attainable value for five paired microorganisms); interpreted as a consistent trend, not as statistical significance. One-sided value (*p* = 0.0313) is reported only as a directional sensitivity analysis and is not a significance claim. Per-microorganism BH-adjusted *p*-values are exploratory and do not override the primary global test.

**Table 6:** Inhibition of biofilm formation by crude and infrared (IR)-treated jojoba oil against the tested bacterial pathogens, expressed as percentage inhibition of adherent biofilm biomass. Biofilm biomass was quantified by crystal violet staining after 24 h of biofilm development in the presence of oil at 25%, 50%, or 75% of the respective MBC. Values are the mean ± standard deviation from three independent experiments, each with six technical replicates; technical replicates were averaged within each independent experiment. Percentage inhibition was calculated as [(A_control_ − A_sample_)/A_control_] × 100 relative to untreated biofilm-growth controls. Blank wells (medium only) served as the negative control. This assay measures inhibition of biofilm formation, not eradication of pre-existing mature biofilms or viable cell counts.

Microorganism	Oil	25% MBC (%)	50% MBC (%)	75% MBC (%)
*Enterococcus faecalis*	Untreated	38.08 ± 1.60	77.64 ± 0.60	84.08 ± 0.40
	IR-treated	79.04 ± 0.50 ***	85.89 ± 0.50 **	92.09 ± 0.90 ***
*Staphylococcus aureus*	Untreated	68.01 ± 0.80	83.76 ± 0.80	91.32 ± 0.30
	IR-treated	79.83 ± 0.60 **	92.16 ± 0.40 **	96.80 ± 0.30 **
*Salmonella typhi*	Untreated	76.63 ± 1.40	89.22 ± 0.20	92.96 ± 0.70
	IR-treated	78.33 ± 0.70 ns	86.76 ± 0.30 **	94.47 ± 0.70 *
*Pseudomonas aeruginosa*	Untreated	53.11 ± 1.10	82.79 ± 1.00	92.10 ± 0.50
	IR-treated	72.59 ± 0.40 ***	91.38 ± 0.50 **	93.59 ± 0.30 *

* Statistical significance was determined by comparing IR-treated jojoba oil with crude jojoba oil at the corresponding concentration: *** *p* < 0.001, ** *p* < 0.01, * *p* < 0.05; ns, not significant (*p* > 0.05). Data are presented as mean ± standard deviation (SD) from three independent experiments.

**Table 7:** Effect of crude and infrared (IR)-treated jojoba oil on the viability of SK-OV-3 human ovarian cancer cells after 24 h of exposure, as determined by the MTT assay.

Concentration (µg/mL)	Untreated Oil Viability (%)	IR-Treated Oil Viability (%)	Adjusted *p*
31.25	99.24 ± 0.20	98.97 ± 0.33	ns
62.5	94.77 ± 0.21	47.66 ± 0.26	***
125	42.05 ± 0.55	19.06 ± 0.23	***
250	6.64 ± 0.23	3.54 ± 0.12	**
500	4.74 ± 0.17	3.43 ± 0.10	**
1000	3.92 ± 0.15	3.27 ± 0.12	*

* Data are presented as mean ± SD from three independent experiments, each performed in triplicate, with technical replicates averaged within each independent experiment. IC_50_ values were estimated using nonlinear regression and compared using the extra sum-of-squares F-test on log IC_50_ values. Concentration-specific comparisons were performed using two-way ANOVA, followed by Sidak’s multiple-comparison test. Adjusted *p* < 0.05 was considered statistically significant. No normal-cell comparator was included; therefore, cancer-cell selectivity and a therapeutic window cannot be established from the present findings. Data are presented as the mean ± SD of biological replicates. Statistical significance is indicated by *** *p* < 0.001, ** *p* < 0.01 and * *p* < 0.05; ns, not significant (*p* > 0.05).

**Table 8:** In fibroblast scratch closure in human dermal fibroblasts (HDFa) following 24 h of treatment with crude or infrared (IR)-treated jojoba oil at a concentration of 125 µg/mL.

Parameter	Untreated Control	Crude Oil (125 µg/mL)	IR-Treated Oil (125 µg/mL)	Adjusted *p*
Initial fibroblast scratch closure width (µm)	1079.34 ± 10.2	1079.34 ± 10.2	1079.34 ± 12.0	ns
Final fibroblast scratch closure width (µm)	355.90 ± 103.8	258.17 ± 138.5	171.37 ± 218.8	*
Scratch closure (%)	67.02 ± 1.8	76.08 ± 4.2	84.12 ± 2.3	*
Calculated migration rate (μm/h)	15.07	17.11	18.92	

Data are presented as mean ± SD from three independent experiments, each comprising two replicate scratches (six replicate scratches in total); technical replicates were averaged within each independent experiment and were not treated as independent observations. IR-treated jojoba oil produced significantly greater scratch closure (84.12%) than crude jojoba oil (76.08%, *p* < 0.01) and the untreated control (67.02%, *p* < 0.05). Data are presented as the mean ± SD of biological replicates. Statistical significance is indicated by * *p* < 0.05; ns, not significant (*p* > 0.05).

**Table 9:** Cell-cycle distribution of SK-OV-3 ovarian cancer cells following treatment with crude or infrared (IR)-treated jojoba oil at their respective IC_50_ concentrations.

Treatment	G0/G1 (%)	S (%)	G2/M (%)
Control	72.5 ± 1.2	25.9 ± 1.0	1.5 ± 0.2
Crude jojoba oil	81.1 ± 1.5 *	17.7 ± 1.2 *	1.05 ± 0.2
IR-jojoba oil	97.9 ± 0.8 **	2.4 ± 0.5 **	0.4 ± 0.1 *

Values are presented as mean ± SD from three independent experiments, each performed in triplicate, with technical replicates averaged within each independent experiment. Cell-cycle percentages were subjected to an arcsine square-root transformation before statistical analysis. Comparisons among the untreated control, crude jojoba oil, and IR-treated jojoba oil groups were performed using one-way ANOVA, followed by Tukey’s HSD post hoc test. Adjusted *p* < 0.05 was considered statistically significant. Data are presented as the mean ± SD of biological replicates. Statistical significance is indicated by ** *p* < 0.01 and * *p* < 0.05.

**Table 10:** Relative mRNA expression of apoptosis-related genes (BCL2, BAX, and CASP3) in SK-OV-3 human ovarian cancer cells after 24 h of exposure to crude or infrared (IR)-treated jojoba oil at their respective IC_50_ concentrations. Values are mRNA fold changes relative to the untreated control and do not represent protein abundance, caspase activation, or functional apoptotic cell death. The BAX/BCL2 ratio is an mRNA ratio only.

Transcript	Control	Crude Oil	IR-Treated Oil	Adjusted *p*
BCL2	1.00 ± 0.04	0.77 ± 0.03	0.61 ± 0.03	*/**
BAX	1.00 ± 0.14	2.11 ± 0.19	3.67 ± 0.23	*/**
CASP3	1.00 ± 0.12	3.16 ± 0.22	5.73 ± 0.30	*/***
BAX/BCL2 mRNA ratio	1.00	2.74	6.02	—

* Adjusted *p* < 0.05 versus control; ** adjusted *p* < 0.01 versus control and crude oil; *** adjusted *p* < 0.001 versus control. Values are the mean ± SD from three independent experiments, each with three technical qRT-PCR replicates; technical replicates were averaged. Fold changes were calculated by the 2^−ΔΔCT^ method after normalization to GAPDH. Data were log_2_-transformed before analysis. Comparisons among control, crude oil, and IR-treated oil were performed by one-way ANOVA followed by Tukey’s HSD per gene. Adjusted *p* < 0.05 was considered significant. These are mRNA-level changes only and do not establish protein abundance, caspase activation, or functional apoptosis.

**Table 11:** Primer sequences used for quantitative real-time PCR analysis.

Gene	Forward Primer (5′→3′)	Reverse Primer (5′→3′)
*BAX*	CCCGAGAGGTCTTTTTCCGAG	CCAGCCCATGATGGTTCTGAT
*BCL2*	GGTGGGGTCATGTGTGTGG	CGGTTCAGGTACTCAGTCATCC
*CASP3*	TGATGGTGACAAGTCAGGGCT	ATCCTGCAAGTGTCAAGGAAG
*GAPDH*	ACAACTTTGGTATCGTGGAAGG	GCCATCACGCCACAGTTTC

## Data Availability

The data supporting the findings of this study are available within the article and its Supplementary Materials; raw data are available from the corresponding author upon reasonable request.

## Supplementary Materials

The following supporting information can be downloaded at: https://www.mdpi.com/article/10.3390/ijms27219415/s1.
