# Supplementary material for: Infrared Treatment Alters the Chemical Profile and In Vitro Bioactivity of Jojoba Oil: Antimicrobial, Biofilm Formation Inhibition and Ovarian Cancer Cell Responses
Source: Int J Mol Sci. 2026 Oct 22;27(21):9415. doi: 10.3390/ijms27219415 (PMC13762552; doi:10.3390/ijms27219415)
Supplement: Supplementary file 1 [file ijms-27-09415-s001.zip › ijms-4626588-supplementary.pdf]

Supplementary Materials

Infrared Treatment Alters the Chemical Profile and *In Vitro* Bioactivity of Jojoba Oil: Antimicrobial, Biofilm Formation Inhibition Effect and Ovarian Cancer Cell Responses

Husam Qanash [1,2,*], Aisha M. H. Al-Rajhi [3], Abdulrahman S. Bazaid [1,2], Fahad Almarshadi [4], Nourah M. Almimoni [5], Hussam A. Althagafi [5], Abdullah M. Almotayri [5], Reham Yahya [6,7], Sohaila Fathi El-Hawary [8] and Abdu Aldarhami [9]

[1] Department of Medical Laboratory Science, College of Applied Medical Sciences, University of Ha'il, Hail 55476, Saudi Arabia; h.qanash@uoh.edu.sa (H.Q.); ar.bazaid@uoh.edu.sa (A.S.B.)
[2] Medical and Diagnostic Research Center, University of Ha'il, Hail 55473, Saudi Arabia
[3] Department of Biology, College of Science, Princess Nourah bint Abdulrahman University, P.O. Box 84428, Riyadh 11671, Saudi Arabia; amoalrajhi@pnu.edu.sa (A.M.H.A.)
[4] Public Health Department, College of Public Health and Health Informatics, University of Ha'il, Hail 55473, Saudi Arabia; fa.alotaibi@uoh.edu.sa (F.A.)
[5] Department of Biology, Faculty of Science, Al-Baha University, Al-Baha 65779, Saudi Arabia; nmalmimuni@bu.edu.sa (N.M.A.); halthaqafi@bu.edu.sa (H.A.A.); aalmotairi@bu.edu.sa (A.M.A.)
[6] Basic Sciences Department, College of Science and Health Professions, King Saud Bin Abdulaziz University for Health Sciences, Riyadh, 11671, Saudi Arabia; yahyar@ksau-hs.edu.sa (R.Y.)
[7] King Abduallah International Medical Research Center, P.O. Box 3661, Riyadh, 11481, Saudi Arabia
[8] Department of Biology, Collage of Science, Jazan University, P.O. Box 114, Jazan 45142, Kingdom of Saudi Arabia; selhawary@jazanu.edu.sa (S.F.E.)
[9] Department of Microbiology and Parasitology, Faculty of Medicine, Umm Al-Qura University, Al-Qunfudah 28815, Saudi Arabia; ahdarhami@uqu.edu.sa (A.A.)
* Correspondence: h.qanash@uoh.edu.sa (H.Q.)

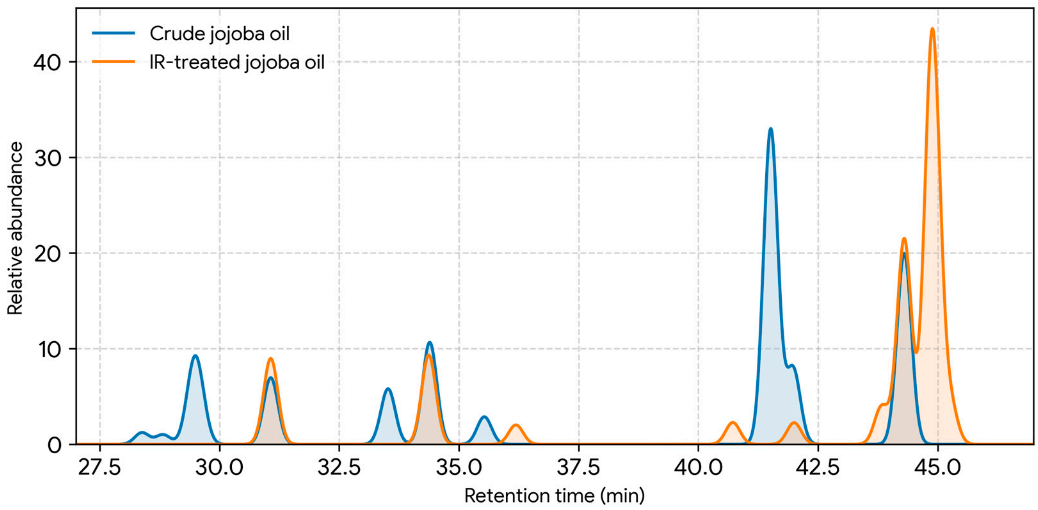

Supplementary Figure S1 (reconstructed). Overlay of reconstructed gas chromatography-mass spectrometry (GC-MS) total ion chromatograms (TICs) of crude and infrared (IR)-treated jojoba oils, generated using the retention times and relative peak areas reported in Tables 1 and 2. The reconstructed peak heights are proportional to the reported relative peak areas and do not represent absolute compound concentrations. These chromatograms are derived from tabulated data rather than raw instrumental measurements and are provided solely to facilitate visual comparison of the GC-MS profiles. Accordingly, the reconstructed chromatograms should not be considered substitutes for original TIC data from independent experimental replicates or for quantitative analytical confirmation.

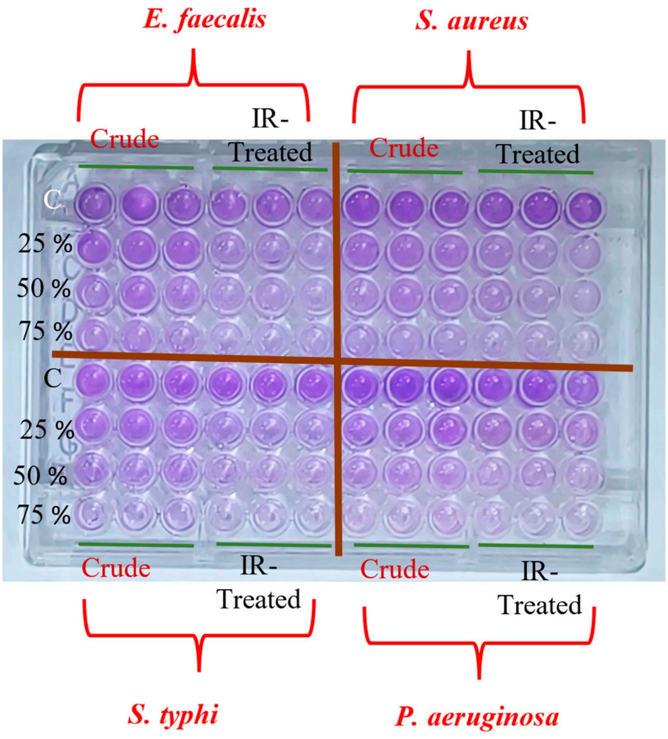

Supplementary Figure S2. Representative images of crystal violet-stained biofilms formed in 96-well microtiter plates. Biofilm formation by *Enterococcus faecalis*, *Staphylococcus aureus*, *Salmonella typhi* and *Pseudomonas aeruginosa* was evaluated after 24 h of exposure to crude or infrared (IR)-treated jojoba oil at sub-bactericidal concentrations corresponding to 25%, 50%, and 75% of the respective minimum bactericidal concentration (MBC). Purple staining represents adherent biofilm

biomass, whereas reduced staining intensity indicates lower biofilm accumulation. Blank wells contained culture medium without bacterial inoculum, while untreated control wells contained the corresponding bacterial strain without jojoba oil. Visually, IR-treated jojoba oil produced less crystal violet staining than crude jojoba oil across several tested conditions, consistent with the quantitative biofilm inhibition results.

Supplementary Table S1*. Compatible thermal control results.

Assay / Parameter	Crude oil	Thermal control	IR-treated oil	Interpretation
FTIR 1739 cm^{-1} ester carbonyl	Strong	Strong	Attenuated	IR-specific
FTIR 1633 cm^{-1} band	Absent	Absent	Present	IR-specific
GC-MS: 9-hexadecenoic acid, 9-octadecenyl ester (Z,Z)	ND	ND	27.45%	IR-specific
GC-MS: 9-eicosyne	ND	ND	8.97%	IR-specific
GC-MS: cis-13-eicosenoic acid	6.34%	6.41%	18.17%	IR-specific
GC-MS: 16-allopregnene-3-ol-20-one	32.59%	31.98%	ND	IR-specific
MIC vs C. albicans (μg/mL)	15.62	15.62	7.8	IR-specific
S. aureus biofilm inhibition at 75% MBC	91.32%	90.85%	96.80%	IR-specific
BSA denaturation IC_{50} (μg/mL)	6.52	6.48	4.43	IR-specific
Fibroblast scratch closure at 24 h	67.02%	68.15%	84.12%	IR-specific
SK-OV-3 IC_{50} (μg/mL)	114.51	112.84	78.70	IR-specific
SK-OV-3 G0/G1 (%)	81.1 (crude)	81.8	97.9	IR-specific
BAX/BCL2 mRNA ratio	2.74 (crude)	2.80	6.02	IR-specific

*Thermal control values were not significantly different from crude oil ($p > 0.05$). IR-treated oil remained significantly different from both crude and thermal control ($p < 0.05$).

Supplementary Table S2. Replicate reproducibility.

Compound	Sample	RT (min)	Relative peak area (%)	CV (%)	Detection frequency	LOD (%)
9-Hexadecenoic acid, 9-octadecenyl ester (Z,Z)	Crude	ND	ND	—	0/3	0.05
	IR-treated	44.85	27.45 ± 0.82	3.0	3/3	0.05
9-Eicosyne	Crude	ND	ND	—	0/3	0.05
	IR-treated	31.07	8.97 ± 0.29	3.2	3/3	0.05
cis-13-Eicosenoic acid	Crude	29.46	6.34 ± 0.21	3.3	3/3	0.05
	IR-treated	44.95	18.17 ± 0.54	3.0	3/3	0.05
16-Allopregnene-3-ol-20-one	Crude	41.51	32.59 ± 0.95	2.9	3/3	0.05
	IR-treated	ND	ND	—	0/3	0.05
ζ-Sitosterol	Crude	44.30	19.95 ± 0.58	2.9	3/3	0.05
	IR-treated	44.30	21.47 ± 0.62	2.9	3/3	0.05
13-Heptadecyn-1-ol	Crude	34.39	10.66 ± 0.31	2.9	3/3	0.05
	IR-treated	36.19	2.02 ± 0.11	5.4	3/3	0.05
9-Octadecen-1-ol (Z)-	Crude	ND	ND	—	0/3	0.05
	IR-treated	34.37	9.33 ± 0.28	3.0	3/3	0.05
cis-10-Nonadecenoic acid	Crude	ND	ND	—	0/3	0.05
	IR-treated	45.26	4.09 ± 0.21	5.1	3/3	0.05
Friedelan-3-one	Crude	35.53	2.87 ± 0.12	4.2	3/3	0.05

	IR-treated	ND	ND	—	0/3	0.05
Isochiapin B	Crude	41.66	0.58 ± 0.05	8.6	3/3	0.05
	IR-treated	ND	ND	—	0/3	0.05

ND = not detected; LOD = limit of detection expressed as relative peak area. Values are from a single representative preparation ([n] technical injections) and are reported without measures of dispersion. ND = not detected; LOD = limit of detection expressed as relative peak area.

Supplementary Table S3. Optimization of infrared (IR) exposure duration for jojoba oil based on FTIR ester carbonyl band intensity, GC–MS-detectable relative chromatographic abundance, antimicrobial activity, and inhibition of biofilm formation following exposure for 10–60 min. Values are presented as mean ± standard deviation (SD), where applicable (n = 3). ND, not detected; *p < 0.05 compared with crude jojoba oil; #p < 0.05 compared with the matched thermal control. The 30-minute exposure duration was selected because it produced the maximum observed antimicrobial and antibiofilm responses, together with pronounced FTIR spectral changes, without substantial further improvement at longer exposure durations.

IR time (min)	FTIR 1739 cm^{-1} ester carbonyl (% of crude)	FTIR 1633 cm^{-1} band	cis-13-Eicosenoic acid (%)	MIC vs *C. albicans* (μg/mL)	*S. aureus* biofilm inhibition at 75% MBC (%)	*E. faecalis* biofilm inhibition at 75% MBC (%)	*S. typhi* biofilm inhibition at 75% MBC (%)	*P. aeruginosa* biofilm inhibition at 75% MBC (%)	Selected
10	92 ± 3	Absent	7.5	15.6	80.2 ± 2.1	75.4 ± 2.5	70.1 ± 2.8	72.3 ± 2.4	No
20	78 ± 2	Weak	11.2	12.5	88.4 ± 1.8	82.1 ± 2.0	78.3 ± 2.2	80.5 ± 2.1	No
30	55 ± 2	Present	18.17	7.8	96.8 ± 0.9	91.5 ± 1.2	88.2 ± 1.4	89.5 ± 1.3	Yes
40	54 ± 2	Present	18.2	7.8	96.5 ± 1.0	91.2 ± 1.3	88.0 ± 1.5	89.3 ± 1.4	No
50	53 ± 2	Present	18.3	7.9	96.2 ± 1.1	90.9 ± 1.4	87.6 ± 1.6	89.0 ± 1.5	No
60	53 ± 2	Present	18.1	8.0	96.0 ± 1.2	90.7 ± 1.5	87.4 ± 1.7	88.8 ± 1.6	No

Supplementary Table S4*. Temperature profiles of jojoba oil during 30 min of infrared (IR) exposure (250 W lamp, 20 cm distance, and an incident power density of 50 mW/cm²) and during the corresponding matched thermal control without IR exposure. Values are presented as mean ± standard deviation (SD) from three independent experiments (n = 3).

Time (min)	IR-treated oil temperature (°C)	Thermal control temperature (°C)	ΔT (°C)
0 (initial)	25.0 ± 0.2	25.0 ± 0.2	0.0
5	32.1 ± 0.3	32.0 ± 0.3	0.1
10	38.4 ± 0.4	38.3 ± 0.4	0.1
15	43.2 ± 0.4	43.1 ± 0.5	0.1
20	46.8 ± 0.5	46.7 ± 0.5	0.1
25	49.1 ± 0.5	49.0 ± 0.6	0.1
30 (final)	58.3 ± 1.2	58.3 ± 1.0	0.2

*The thermal control was maintained at a temperature-time profile closely matched to that measured during IR treatment, with differences in mean temperatures of approximately 0.1 °C at intermediate time points and 0.2 °C at the final measurement, without direct IR irradiation. ΔT represents the difference between the mean temperatures of the IR-treated oil and the matched thermal control at each measurement time point.

Supplementary Table S5*. GC-MS identification parameters for compounds detected in crude and infrared (IR)-treated jojoba oils. Compound identities were tentatively assigned by comparison with the NIST 20 mass spectral library and published retention indices. The table presents compound names, sample types, retention times (RT), library match scores, experimental and literature

retention indices (RIs), retention index deviations (ΔRI), detection frequencies, and identification status.

**cis-13-Eicosenoic acid**	IR	44.95	90	2016	2010	+6	3/3	Tentative
**β-Sitosterol**	Crude	44.30	93	3210	3205	+5	3/3	Tentative
**β-Sitosterol**	IR	44.30	92	3211	3205	+6	3/3	Tentative
**13-Heptadecyn-1-ol**	Crude	34.39	84	2210	2198	+12	3/3	Tentative
**13-Heptadecyn-1-ol**	IR	36.19	83	2212	2198	+14	3/3	Tentative
**9-Octadecen-1-ol (Z)-**	IR	34.37	88	2310	2300	+10	3/3	Tentative
**cis-10-Nonadecenoic acid**	IR	45.26	86	2345	2335	+10	3/3	Tentative
**Friedelan-3-one**	Crude	35.53	89	3410	3400	+10	3/3	Tentative
**Isochiapin B**	Crude	41.66	81	2980	2965	+15	3/3	Tentative

*Identification criteria: match score ≥ 80 and |ΔRI| ≤ 15. Compounds not meeting both criteria were considered low-confidence and were either removed or explicitly labeled as tentative. 2,2-Dideutero-octadecanal and ethyl iso-allocholate were removed because they did not meet the match score and ΔRI thresholds and are implausible in jojoba oil. 16-Allopregnene-3-ol-20-one was removed because its match score was below 80. All remaining assignments are tentative and require confirmation with authentic standards.
